# A posttranslational proteomic survey of a single anatomically preserved human 20‐week postconception brain

**DOI:** 10.1111/joa.70170

**Published:** 2026-05-03

**Authors:** S. Bandiera, H. Bogetofte, P. Jensen, R. Hussain, S. A. Nischal, L. Rihova, G. J. Clowry, M. R. Larsen, Z. Molnár, B. C. Carlyle

**Affiliations:** ^1^ Department of Physiology, Anatomy & Genetics University of Oxford Oxford UK; ^2^ Department of Biochemistry and Molecular Biology University of Southern Denmark Odense M Denmark; ^3^ Newcastle University Biosciences Institute and Centre for Transformative Neuroscience Newcastle upon Tyne UK; ^4^ Kavli Institute for Nanoscience Discovery University of Oxford Oxford UK

**Keywords:** brain development, development, posttranslational modifications, proteomics, thalamocortical connections

## Abstract

Progress in defining the proteome of the developing human brain has lagged behind our understanding of the adult human brain, primarily due to challenges in tissue acquisition and in preservation of anatomical structure during experimental processing. Single‐cell transcriptomics alone is an excellent resource for defining cellular identity, but has limited capacity to trace neuronal connectivity because proteins, the active molecules in interactions, may be transported significant distances from cell bodies and their site of synthesis. There are numerous protein‐mediated transient interactions between cellular elements in the developing brain, such as between migrating cortical neurons and subplate, and thalamic projections and cortical progenitors. Anatomical approaches have identified specific cell populations that interact, allowing us to characterize the transient and dynamically changing early circuits. Proteomic data generation is now essential for ligand–receptor pair prediction and validation. Upon receipt of a single, exceptionally well‐preserved 20 postconception week human brain hemisphere, we conducted fine dissections of 18 anatomically distinct brain regions, including the pia mater. These samples underwent in‐depth analysis of both the total and posttranslationally modified proteomes, with the aim of creating a reference resource for investigators studying this critical stage of neurodevelopment. Here, we have presented an overview of the resulting dataset, compared the proteomic profiles across regions, and highlighted examples of variable posttranslational modifications within individual proteins. As expected, non‐modified protein profiles revealed substantial differences across brain regions and structures. For instance, pia mater and thalamus were enriched for proteins involved in transcription and chromatin organization, which may suggest a higher proportion of dividing cells and/or significant epigenetic regulation in these areas at this developmental stage. In contrast, the cortical and hippocampal proteome reflected active synaptogenesis and cytoskeletal remodeling. While interregional differences in phosphorylated and acetylated peptides largely mirrored those observed in the non‐modified proteome with respect to gene ontology categories, the glycosylated peptidome of the pia mater was markedly distinct. This divergence is driven by the secretion of extracellular matrix proteins and the region's intimate association with the basement membrane of the pia. Finally, by integrating our proteomic data with publicly available single‐cell RNA sequencing datasets from the same developmental stage, we identified high‐confidence ligand–receptor pairs (e.g., L1CAM:CD9, CNTN4:PTPRG, LGALS1:ITGB1) likely involved in thalamocortical interactions.

## Graphical abstract

A well‐preserved 20 post‐conception week human brain was obtained and finely dissected into 18 anatomically distinct regions, including the pia mater. Each region underwent in‐depth proteomic analysis, encompassing both total protein content and post‐translational modifications. This approach generated a spatially resolved proteomic resource of the 20 PCW human brain for downstream comparative and integrative analyses.

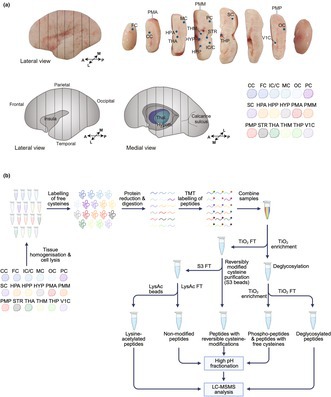

## 1 INTRODUCTION

Many of the developmental processes in the brain are not understood because we do not integrate methods that can reveal mechanisms from molecules to connectivity. Single‐cell and spatial transcriptomics have revolutionized our understanding of cellular identity and diversity, but these methods can only reach their potential if we integrate them with detailed cellular anatomy that informs on the specific cellular interactions and interconnections. Our hypothesis is that transient synapses in the developing brain have unique structure and molecular mechanisms of protein transport, release and neurotransmission that are different from the adult mature brain. Analysis of protein abundance and regional location is therefore key to validating receptor–ligand interactions from single‐cell transcriptomic datasets. The distribution of proteins, particularly when secreted and acting at locations distant from the site of transcription, is key to understanding transient cellular and molecular interactions in the developing brain. Analysis of posttranslational modifications (PTMs) such as phosphorylation and glycosylation will shed further light on the state of intracellular signaling pathways downstream of these interactions, and secretion and modification of extracellular facing proteins. We believe that by combining anatomy, transcriptomics, and proteomics, we can provide a new understanding of cell–cell interactions in the developing brain.

The primary aim of this single‐brain study was to provide both a proof of concept and a resource for researchers investigating protein expression across anatomical regions of the developing human brain at 20 postconception weeks (PCW). Specifically, the dataset enables exploration of both total protein abundance and the extent of PTMs associated with proteins of interest. Derived from a single anatomically well‐preserved 20 PCW human brain, this dataset serves to demonstrate the utility of PTM‐resolved proteomics in developmental neuroscience and encourage broader adoption of these techniques in future, higher powered studies.

To date, comprehensive proteomic profiling of the human fetal brain remains limited. Djuric et al., performed label free mass‐spectrometry (MS) on multiple brain regions from 8 FFPE fixed human brain tissue at selected timepoints from 16 to 36 gestational weeks (GWs). They quantified approximately 3000 proteins and showed that samples clustered by domain/zone rather than developmental stage. The ventricular zone was distinctly separated from other regions across all timepoints, being dominated by gene ontology (GO) terms such as DNA replication, as opposed to synaptic proteins in other regions (Djuric et al., 2017). In 2022, Zhao et al., quantified approximately 2500 proteins in three human brain samples from 9, 11, and 13 GWs, identifying a small number of proteins that changed abundance between developmental stages, including three proteins associated with neurogenesis (Zhao et al., 2022). Nascimento et al. (2019) employed label‐free shotgun proteomics to characterize human stem cell‐derived cerebral organoids, identifying 3073 proteins across various developmental stages and cell types, including neural progenitors, neurons, astrocytes, and oligodendrocytes. More recently, Melliou et al. (2022) performed comparative proteomic analysis between precursor and neuronal cell populations in cerebral organoids and mid‐gestation human brain tissue using mass spectrometry. This study marked a significant step forward, particularly considering earlier transcriptomic comparisons (Camp et al., 2015; Carlyle et al., 2017; Quadrato et al., 2017; Velasco et al., 2019) frequent discrepancies between RNA and protein abundance in both developing brains and in vitro models.

In primates, Wei et al. (2023) generated a spatiotemporal proteomic atlas of the cynomolgus macaque (*Macaca fascicularis*) brain, profiling multiple regions across four developmental stages from early fetal to neonatal. In contrast to Djuric et al., but agreement with human brain transcriptomics (Lindsay et al., 2016), they found that developmental stage contributed more to proteomic variability than brain region and identified region‐specific protein dynamics. This disagreement may be a reflection of the lower proteomic depth and potential bias in extracting proteins from FFPE tissue in the human study, and/or the choice of brain regions sampled in each study. Low‐resolution comparison of frozen versus FFPE fixed colon adenoma samples showed a moderate decrease in protein identifications in FFPE tissue, with no significant difference in gene ontology categories represented by the proteins detected (Sprung et al., 2009). Using higher resolution proteomics, Schoffman et al., showed no difference in the number of identified peptides and proteins in fixed versus frozen meningioma samples. They did detect significant differences in the extent of PTMs, with FFPE samples producing more formylated and methylated peptides, while frozen tissues had more phosphorylated peptides. FFPE samples had a higher missed cleavage rate than frozen samples, and samples clustered by their preservation method, rather than by individual. Proteins with a high molecular weight were over‐represented in frozen tissue compared to fixed, and effect also observed in another publicly available dataset (Schoffman et al., 2022). Plantera et al., compared fresh frozen, FFPE and formalin fixed brain tissue, obtaining more protein identifications from frozen tissue than fixed, with few unique protein detections in fixed samples. Frozen tissue was clearly separated from fixed tissue by principal components analysis. Fixed samples showed depletion in plasma and mitochondrial membrane proteins, with an enrichment for synaptic proteins and better retention of hydrophobic peptides in FFPE samples (Plantera et al., 2026).

Given spatially resolved datasets are lacking for the human brain, direct cross‐species comparisons are limited. To address this gap, Wang et al. (2023) profiled the proteomic landscape of enriched postsynaptic densities (PSDs) in the prefrontal cortex (PFC) across species and developmental stages. Their analysis revealed a markedly slower maturation of human PSDs, highlighting species‐specific aspects of synaptic development. While this study featured a larger sample size and temporal resolution, it was restricted to the PFC and more specifically to PSDs, leaving other cortical and subcortical regions, as well as additional subcellular compartments, unexplored.

Although PTMs are well established as critical modulators of protein function across diverse biological systems, including the nervous system (Mann & Jensen, 2003; Ren et al., 2014; Smith & Carregari, 2022; Suskiewicz, 2024; Zhong et al., 2023), they have not been systematically characterized in existing proteomic datasets of the developing brain. Transcript detection does not guarantee protein expression, and protein presence does not confirm it is functionally active in the absence of relevant PTMs. We have previously shown that high‐resolution mass spectrometry, combined with a multi‐step enrichment strategy, can reliably quantify peptides modified by phosphorylation, reversible cysteine modification, and N‐linked glycosylation from the same biological sample (Bogetofte et al., 2023; Elmkvist et al., 2024; Kang et al., 2018).

In this study, we present quantitative data from both non‐modified proteins and posttranslationally modified peptides across 18 distinct brain regions of a single 20 PCW human brain. We provide illustrative examples to demonstrate the utility of the dataset for investigating regional proteomic variation and modification‐specific biology. Through this work, we aimed to emphasize the value of PTM‐resolved proteomics and advocate for its application in future large‐scale, multi‐sample, multi‐timepoint developmental studies.

## 2 METHODS

### 2.1 Sample source

The left hemisphere of a human fetal brain was obtained from a medical abortion at 20 PCW by the MRC/Wellcome Trust funded Human Developmental Biology Resource (HDBR), University of Newcastle. The tissue was collected with maternal consent and approval from the Newcastle and North Tyneside NHS Health Authority Joint Ethics Committee. The hemisphere was flash‐frozen upon collection and shipped in dry ice according to the material transfer agreement (H‐20068526) between the University of Newcastle and the University of Oxford. The sample was screened for the absence of major brain abnormalities and common genetic defects by HDBR personnel before selection for this investigation.

### 2.2 Dissection

**FIGURE 1:**
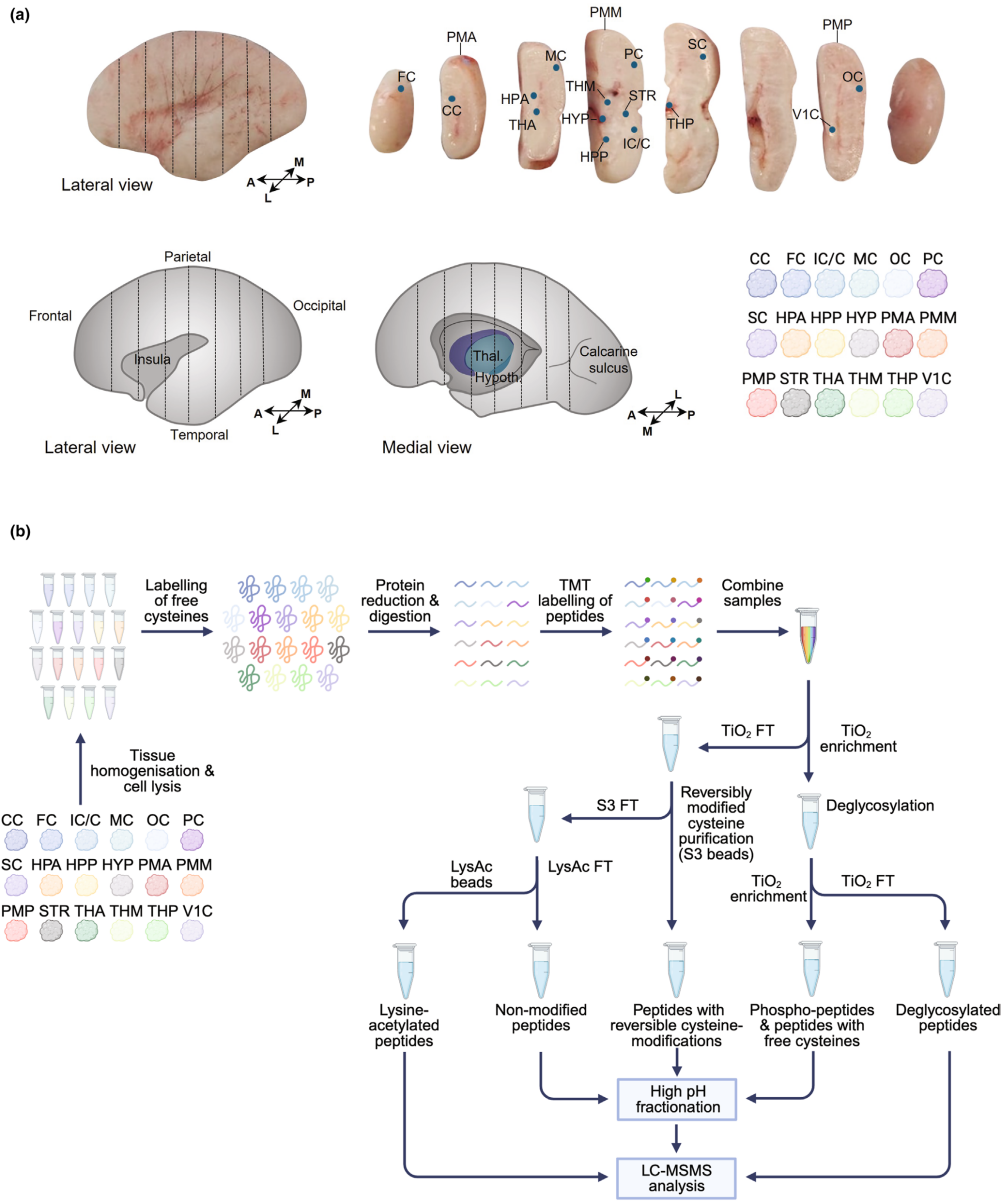
Experimental overview. (a) Photographs and schematic diagrams of the sample showing slab locations and dissected regions within each slab. (b) Workflow for the enrichment and analysis of posttranslational modifications by liquid chromatography tandem mass spectrometry (LC‐MSMS) proteomic analysis. The 18 fetal brain region samples were homogenized and lysed prior to labelling of free cysteines with CysPAT. The proteins were reduced and digested before labelling of peptides with isobaric Tandem Mass Tags (TMT) for relative quantification. Equal amounts of tagged peptides from each sample were combined and titanium dioxide (TiO_2_) enrichment performed to isolate phospho‐peptides, sialylated N‐linked glycopeptides and free cysteine‐containing peptides (labelled with CysPAT). Following deglycosylation a second TiO_2_ enrichment was performed to isolate the glycopeptides. From the first TiO_2_ flow‐through (FT) S3 thiol capture beads and anti‐Lysine‐acetyl antibody conjugated beads were used to isolate peptides with reversible cysteine modifications and lysine acetylation, respectively, from the non‐modified peptides. High pH fractionation was performed to increase coverage of the samples with the most material.

The hemisphere was kept frozen during dissection. Eight coronal slabs approximately 0.5–1 cm thick were manually dissected with a long surgical blade (Figure 1a). Regions of interest were then microdissected using surgical instruments following major anatomical landmarks. The location of each dissected region is indicated in Figure 1a. To ensure consistency, all dissections were carried out by ZM with the help of SB. Samples were collected in clean tubes and immediately stored at −80°C until further processing. Region abbreviations can be found in Table 1.

**TABLE 1:** Region abbreviation terms used throughout the manuscript, and corresponding Brainspan region for RNA comparisons.

Abbreviation	Region	Brainspan equivalent region
CC	Cingulate Cortex	Anterior cingulate cortex
FC	Frontal Cortex	Dorsolateral prefrontal cortex
IC/C	Insular Cortex/Claustrum	N/A
MC	Motor Cortex	Primary Motor Cortex
OC	Occipital Cortex	N/A
PC	Parietal Cortex	Posteroventral Parietal Cortex
SC	Somatosensory Cortex	Primary Somatosensory Cortex
HPA	Anterior Hippocampus	Hippocampus
HPP	Posterior Hippocampus	Hippocampus
HYP	Hypothalamus	N/A
PMA	Anterior Pia Mater	N/A
PMM	Medial Pia Mater	N/A
PMP	Posterior Pia Mater	N/A
STR	Striatum	Striatum
THA	Anterior Thalamus	N/A
THM	Medial Thalamus	Mediodorsal Nucleus of Thalamus
THP	Posterior Thalamus	N/A
V1C	Primary Visual Cortex	Primary Visual Cortex

### 2.3 Tissue sample preparation

Samples were lysed by two 30‐s rounds of mechanical homogenization in a bead beater (Stretton Scientific) in pH 8 lysis buffer (5% w/v sodium deoxycholate (SDC, Sigma #D6750) in 100 mM HEPES (Merck #1003378397) with added cOmplete protease inhibitor (Merck #11836170001) and PhosSTOP phosphatase inhibitor (Merck #4906837001) and kept on ice between homogenization rounds). Samples from the pia were lysed in 300 mL of lysis buffer; all other samples were lysed in 500 mL. Following homogenization, samples were centrifuged in a desktop centrifuge at 2000 rpm for 5 min. The supernatant was removed to a new Eppendorf tube (SafeSeal low protein binding, Sarstedt #72.706.600) before sonication for 2 × 20 s using a probe sonicator on Setting 2 (XL‐2000 Ultrasonic Liquid Processor, Misonix). After sonication, samples were heated at 95°C for 5 min, before centrifuging at 17,000*g* at 4°C for 10 min. Supernatants were removed to a new Eppendorf tube and placed in a SpeedVac at room temperature for up to 4 h. After Speed Vac, the samples appeared “jelly‐like,” and were stored at −20°C prior to shipping.

Samples were shipped on dry ice. Upon receipt, frozen sample pellets were resuspended in 5% SDC/HEPES (100 mM) buffer (pH 8.5) sonicated at 60% amplitude for 2 × 20 s using a Q125 probe‐sonicator (QSONICA) and subsequently centrifuged for 20 min at 20,000*g* to pellet insoluble material. Protein concentrations were measured using a Implen NanoPhotometer N60 (Implen, Germany). A total of 150 μg protein from each sample was added to Amicon Ultra‐0.5 10 kDa Centrifugal filters (Merck Millipore #UFC201024) and diluted to 200 μL in 10 mM ammonium acetate (Sigma‐Aldrich #73594) (pH 7.5). The spin filters were centrifuged for 20 min at 11,000*g* to pass the solution through. Proteins on top of the filters were diluted in 100 μL 100 mM HEPES buffer (pH 8.5).

### 2.4 Labelling of free cysteines

The Cysteine‐specific Phosphonate Adaptable Tag (CysPAT) was prepared as previously described (Huang et al., 2016). The CysPAT solution was added to proteins on the filters to a final concentration of 5 mM CysPAT and left to react with the free cysteine residues in the proteins for 1 h at 37°C, resulting in N‐succinimidyl iodoacetate (SIA) modification. After incubation, the solution was passed through the filter, which was washed twice using a 100 mM HEPES buffer containing 0.2% SDC (pH 8.5).

### 2.5 Reduction and digestion

Reduction of proteins on the filters was performed by 30 min incubation with 3 mM tris(2‐carboxyethyl)phosphine (TCEP, Sigma‐Aldrich #646547) in 1% SDC/100 mM HEPES (pH 8.5). Digestion of proteins was achieved using lysyl endoproteinase (0.00004 AU per μg protein; Wako #129‐02541) and 5% (w/w) dimethylated trypsin (Heissel et al., 2019) overnight (ON) at 37°C. Next day, samples were incubated at 37°C for 1 h with another 1% (w/w) dimethylated trypsin. The digested peptides were transferred to a low‐binding Eppendorf tube, and 30 μL 30% acetonitrile (VWR #83640.290) was used to wash the filter and pooled with the peptide digest.

### 2.6 TMT labelling

Peptide concentration was measured using the Nanodrop and 100 μg peptide in 100 μL HEPES with 1% SDC (pH 8.5) from each sample was labelled with TMTpro 18‐plex (Thermo Fisher Scientific #A52045) for 1.5 h. A total of 1 μL of each sample was combined and analyzed by LC–MS/MS to ensure proper labelling of all TMT channels. Subsequently, samples were pooled in a 1:1 ratio, and excess reagent was quenched by incubation for 15 min at RT with 10 μL 1 M ammonia bicarbonate (Sigma‐Aldrich #A6141). Following this, the pooled TMT sample was acidified with 2% formic acid (Merck #1.11670.0250) and vortexed to pellet the SDC. The samples were centrifuged at 20,000*g* for 15 min and the supernatant was transferred to a new low‐binding tube and dried by vacuum centrifugation to a volume of 150 μL.

### 2.7 Enrichment of phosphopeptides, sialylated N‐linked glycopeptides, and free cysteine‐containing peptides (SIA peptides)

The combined sample was diluted to 1 mL with 5% trifluoroacetic acid (TFA, Sigma‐Aldrich #S6046378), 1 M glycolic acid (Fluka #50590), and 80% acetonitrile and incubated under gentle rotation for 10 min with 10 mg of TiO_2_ beads (GL Science #5020‐75,000). After incubation, the sample was centrifuged for 15 s in a tabletop centrifuge to pellet the TiO_2_ beads. The supernatant was transferred to another low‐binding Eppendorf tube and incubated with 5 mg TiO_2_ beads under gentle rotation at RT for 10 min. The sample was then centrifuged in a desktop centrifuge and the supernatant recovered to a new Eppendorf tube with unbound peptides. The TiO_2_ beads from both incubations were pooled with 200 μL 1% TFA, 80% acetonitrile in a new Eppendorf tube and after mixing and centrifugation, the supernatant was added to the first supernatant. This supernatant (A) was lyophilized by vacuum centrifugation. The washed TiO_2_ pellet was dried for 10 min by vacuum centrifugation and resolubilized in 150 μL 100 mM HEPES (pH 8.5). The sample was incubated with PNGaseF (New England Biolabs #P0705L) and Sialidase A (Agilent Technologies #GK80040) at 37°C ON. The next day, the TiO_2_‐enriched peptides were reduced by incubation with 5 mM dithiothreitol (DTT, Sigma #D9162) for 20 min at room temperature and alkylated with 10 mM iodoacetamide (IAA, Sigma #l1149) for 30 min at RT. Following this, the sample was diluted to 1% TFA/70% acetonitrile and incubated under rotation for 10 min to allow the phosphopeptides and SIA peptides to reattach to the TiO_2_ beads. The sample was then centrifuged at 14,000*g* to pellet the TiO_2_ beads and the supernatant containing deglycosylated N‐linked glyco‐peptides (B) was lyophilized by vacuum centrifugation. The TiO_2_ pellet was incubated in 150 μL 5% ammonia under rotation for 10 min at RT to allow efficient elution of phosphopeptides and SIA peptides. The TiO_2_ beads were pelleted by centrifugation and the supernatant (C) run through a p200 tip with a C8 material from a 3 M EmporeTM disk (Sigma #66882‐U) to capture TiO_2_ beads. The solution was recovered in a low‐binding Eppendorf tube. A total quantity of 100 μL 5% ammonia/50% acetonitrile was mixed with the TiO_2_ beads. Following centrifugation, the supernatant was run through the same p200 tip/C8 disc and collected with the initial elution (C) prior to lyophilization by vacuum centrifugation.

### 2.8 Enrichment of cysteine‐containing peptides with reversible modifications

The first supernatant (A) from the TiO_2_ enrichment was resolubilized in 2 mL 0.1% TFA (pH < 2) and desalted using a 10 mg Oasis HLB cartridge (Waters #186000383). After washing with 0.1% TFA, the peptides were eluted with 1 mL 60% acetonitrile in water and lyophilized. The peptide solution was resolubilized in 50 μL 500 mM HEPES (pH 8.5) and incubated with 5 mM TCEP for 1 h at RT. Three hundred microliter of Agarose S3 high‐capacity acyl‐rac capture beads (NANOCS Inc. #AR‐S3‐2) were washed once in 100 mM HEPES (pH 8.5). The peptide solution was diluted to 1.2 mL in H_2_O and incubated with the S3 beads for 1 h with rotation at RT. The beads were then pelleted by gentle centrifugation using a tabletop centrifuge and the supernatant was recovered to a low‐binding Eppendorf tube (D). The S3 beads were washed with 300 μL 100 mM HEPES (pH 8.5), centrifuged gently and the supernatant was added to the Eppendorf tube (D). The S3 beads were solubilized in 500 μL H_2_O, transferred to a MobiSpin column filter (10 μm, MoBiTec Molecular Biology #M105010S), and washed three times with 500 μL H_2_O. The S3 beads were incubated with 300 μL 100 mM HEPES (pH 8.5) with 10 mM DTT at RT for 30 min to release the cysteine‐containing peptides. The solution containing the reversibly modified cysteine‐containing peptides (E) was passed through the MobiSpin column filter, collected in a new low‐binding Eppendorf tube, alkylated with 20 mM IAA for 30 min at RT in the dark, and finally lyophilized by vacuum centrifugation.

### 2.9 Enrichment of lysine‐acetylated peptides

The supernatant (D) was adjusted to pH 7.2 using hydrochloric acid (HCl, Merck #1003180500) and incubated with 20 μL Immunoaffinity beads carrying Anti‐Lysine‐acetyl antibodies conjugated to Agarose beads (PTM‐Scan^â^ Acetyl‐Lysine Motif [Ac‐K], Cell Signaling Technology #13362) for 2 h at RT with gentle rotation. Afterward, the beads were pelleted by gentle centrifugation and the supernatant containing non‐modified peptides (F) transferred to a new low‐binding Eppendorf tube. The beads were washed using the buffer in the PTMScan kit three times and the lysine‐acetylated peptides were eluted by incubation with 150 μL 0.15% TFA for 10 min. The beads were pelleted, and the supernatant transferred to another low‐binding Eppendorf tube (G). The beads were washed with 100 μL 0.15% TFA, which was added to the eluate (G) and lyophilized prior to LC–MS/MS.

### 2.10 High pH fractionation

Samples containing phospho‐peptides/SIA peptides (free cysteines) (C), peptides with reversibly modified cysteines (E), and the non‐modified peptides (F) were separately dissolved in 20 mM ammonium formate, pH 9.3, loaded on an Acquity UPLC®—Class CSHTM C18 column (Waters) and fractionated on a Dionex Ultimate 3000 HPLC system (Thermo Scientific) into 20 concatenated fractions each based on UV detection at 210 nm. The fractions were lyophilized by vacuum centrifugation prior to LC–MS/MS.

### 2.11 Nano‐flow liquid chromatography–mass spectrometry (nLC‐MS/MS)

#### 2.11.1 Non‐modified peptide fraction

The non‐modified peptide fractions from the High pH RP separations were resolubilized in 6 μL 0.1% formic acid (Buffer A) and analyzed by SPS‐RT‐MS using the Orbitrap Eclipse Tribrid MS instrument (Thermo) (Schweppe et al., 2020). Peptides were loaded onto an in‐house made fused silica capillary 20 cm × 100 μm inner diameter packed with Reprosil‐Pur 120 C18‐AQ, 1.9 μm (Dr. Maisch GmbH) on an EASY‐nLC system (Thermo Scientific) coupled to an Orbitrap Eclipse Tribrid MS instrument. The peptides were eluted using increasing buffer B (95% ACN, 0.1% FA) from 2 to 22% in 110 min, from 22% to 40% B for 30 min, and from 40% to 95% B for 1 min. MS1 spectra were recorded for *m*/*z* range 350–1600 using 120.000 FWHM resolution using an AGC value of 250% and maximum filling time of 50 ms. Peptides were selected for 3 s cycle time for MS/MS using isolation window of 0.7 Da and peptides were fragmented using CID (NCE 35) in the linear ion trap using turboscan. Each CID low‐resolution spectrum was searched using real‐time searching in the building database software using the following settings: the human Uniprot fasta database (version from 02.06.2023; 42,252 sequences); TMTpro (K and N‐terminal) as fixed modifications; mass accuracy 10 ppm for MS1 and 20 ppm for MS2. For the precursors who matched a sequence in the MS2 search, the precursor was reselected and fragmented and 10 fragment ions were selected by synchronous precursor selection (SPS) for HCD fragmentation (NCE 55), and the TMT reporter ions were recorded in the orbitrap with TurboTMT settings.

### 2.12 Phosphopeptide and SIA peptide fraction

All high pH RP fractions containing phosphopeptide and SIA peptides (free cysteines) were dissolved in 3.5 μL buffer A (0.1% FA) and analyzed by nano LC‐ESI‐MS/MS using an EASY‐nLC (Thermo Fisher Scientific) with buffer A (0.1% FA) and buffer B (95% ACN, 0.1% FA) connected online to an Orbitrap Exploris™ 480 mass spectrometer (Thermo Fisher Scientific, US). The peptides were loaded onto an in‐house made pulled emitter analytical column (20 cm × 100 μm inner diameter packed with Reprosil‐Pur 120 C18‐AQ, 1.9 μm [Dr. Maisch GmbH]). The peptides were eluted using increasing buffer B (95% ACN, 0.1% FA) from 2% to 30% in 100 min and then from 30% to 50% B for 20 min. For each cycle, a full MS spectrum was obtained in the mass range of *m*/*z* 350–1500 in the Orbitrap Explore 480 with a resolution of 120,000 FWHM, a maximum injection time of 50 ms, and an AGC of 300%. Hereafter, the peptides were automatically selected for MS/MS for a 3 s cycle time using isolation window of 0.7 Da, HCD with NCE setting at 33, resolution of 45,000 FWHM, AGC target value of 300% and maximum injection time of 100 ms.

### 2.13 Reversibly modified cysteine fraction

All high pH RP fractions containing reversibly modified cysteine (RmCys) peptides were dissolved in 3.5 μL buffer A (0.1% FA) and analyzed by nano LC‐ESI‐MS/MS using an EASY‐nLC (Thermo Fisher Scientific) with buffer A (0.1% FA) and buffer B (95% ACN, 0.1% FA) connected online to an Orbitrap Exploris™ 480 mass spectrometer (Thermo Fisher Scientific, US). The peptides were loaded onto an in‐house made pulled emitter analytical column (20 cm × 100 μm inner diameter packed with Reprosil‐Pur 120 C18‐AQ, 1.9 μm [Dr. Maisch GmbH]). The peptides were eluted using increasing buffer B (95% ACN, 0.1% FA) from 2% to 25% in 75 min and then from 25% to 40% B for 15 min. For each cycle, a full MS spectrum was obtained in the mass range of *m*/*z* 350–1400 in the Orbitrap Explore 480 with a resolution of 120,000 FWHM, a maximum injection time of 50 ms, and an AGC of 300%. Hereafter, the peptides were automatically selected for MS/MS for a 3 s cycle time using isolation window of 0.7 Da, HCD with NCE setting at 33, resolution of 45,000 FWHM, AGC target value of 300%, and maximum injection time of 100 ms.

### 2.14 Deglycopeptides and lysine acetylated peptides

Both peptide fractions were analysed directly using a two‐column system on an nEASY‐LC system, where the peptides were first loaded onto a home‐made pre‐column (2 cm) containing Reprosil‐Pur 120 C18‐AQ, 3 μm (Dr. Maisch GmbH). After washing the pre‐column, the peptides were eluted onto a 20 cm × 100 μm inner diameter packed with Reprosil‐Pur 120 C18‐AQ, 1.9 μm (Dr. Maisch GmbH) into an Orbitrap Eclipse Tribrid MS instrument. The peptides were eluted using increasing buffer B (95% ACN, 0.1% FA) from 2% to 22% in 100 min and then from 22% to 40% B for 40 min. For each cycle, a full MS spectrum was obtained in the mass range of *m*/*z* 300–1500 in the Orbitrap with a resolution of 120,000 FWHM, a maximum injection time of 50 ms, and an AGC of 250%. Hereafter, the peptides were automatically selected for MS/MS for a 3 s cycle time using an isolation window of 0.7 Da, HCD with NCE setting at 34, a resolution of 50,000 FWHM, an AGC target value of 100%, and a maximum injection time of 100 ms.

### 2.15 Peptide/protein identification and quantification

The raw files obtained from the non‐modified peptides were searched in Proteome Discoverer (PD, v2.5; Thermo Scientific) using the Sequest HT search engine against the Human Uniprot fasta database (version June 2023; 48,640 sequences). The following search parameters were used for the database searches in PD 2.5: precursor mass tolerance 10 ppm, fragment mass tolerance 0.8 Da (SPS‐RT‐MS3 data), and trypsin digestion with a maximum of two missed cleavages. All searches were performed with TMTpro (K) and TMTpro(N‐term) as static modifications. Peptide identifications were filtered against a 1% false discovery rate cutoff using the Percolator (Spivak et al., 2009) algorithm. For the non‐modified peptide dataset, peptide identifications were assembled into protein groups. Quantification was performed based on the intensities of the TMT reporter ions from the MS3 data.

The datasets from phosphopeptide/SIA peptide fractions, reversibly modified cysteine fractions, and deglycopeptide fractions were all searched in PD 2.5 using first the Mascot database search algorithm, and the MS2 spectra that did not match anything in Mascot were subsequently searched in the SEQEUST database search algorithm. The Human Uniprot fasta database (version June 2023, 48,640 sequences) was used for all searches. Precursor mass tolerance for MS was 10 ppm, fragment mass tolerance was 0.05 ppm, and a maximum of two missed cleavages was used. In all searches, TMTpro (K) and TMTpro (N‐term) were set as static modifications. Dynamic carbamidomethyl (C) and SIA (CysPAT free cysteines) were used in all searches, and phosphorylation of serine, threonine, and tyrosine, and deamidation on N were set as dynamic modifications in the phosphopeptide/SIA and deglycopeptide analyses. For the database searching of the lysine acetylated peptide raw data, TMTpro (K) was variable together with variable lysine acetylation (K), and a maximum of four missed cleavages was used.

### 2.16 Downstream analysis

Following protein and peptide identification and quantification, quantification tables were read into R Studio (v 1.3.1056). Uniprot IDs were converted to Gene symbols using a reference downloaded from Ensembl Biomart. Common contaminants, including keratins, were removed using a published list (Frankenfield et al., 2022). For non‐modified proteins, a threshold of two unique peptides was required for inclusion in further analysis. Downstream analysis and visualization were performed using base R (v 4.0.0.2) and tidyverse packages (v 1.3.0). GO analysis was performed using String‐db.org webtools using default settings with the “multiple proteins” search tool. tsv files were downloaded from string and used for plotting in R. The Statistical background set used for all these enrichment analyses was all proteins detected in our study.

### 2.17 RNA–protein correlations

The regional RNAseq data matrix and metadata were obtained from https://www.brainspan.org/static/download.html. RNAseq data were available from 19 PCW and 21 PCW; the Pearson correlation between normalized read counts for these two timepoints was 0.91, and so only the data from the 19 PCW were used for comparison, as there were fewer missing values in this dataset. See Table 1 for region matching.

### 2.18 Ligand–receptor pair prediction

Raw single cell RNAseq (scSEQ) count matrices for PCW20 were accessed directly from https://data.nemoarchive.org/biccn/grant/u01_devhu/kriegstein/transcriptome/scell/10x_v2/human/processed/counts/ (Bhaduri et al., 2021). Using the Seurat R package (v4.3.0 [Hao et al., 2021]), data were filtered to retain cells with between 300 and 3000 features and with mitochondrial DNA less than 10%. After normalization with the “NormalizeData” function, and identification of the top 2000 variable genes with the “FindVariableFeatures” function, the data were scaled to standardize gene expression across cells using the “ScaleData” function. Principal component analysis (PCA) was performed, selecting eight PCs for the thalamus and 12 PCs for the PFC datasets. Cells were clustered using the “FindNeighbours” and “FindClusters” functions. Cell types were annotated based on known gene markers: mature thalamic projection neurons (*L1CAM, SLC17A, VGLUT2, TCF7L2, EFNA5, VGF, CALB2, NTNG1, ACHE*) (Gerstmann et al., 2015; Krsnik et al., 2017; Monko et al., 2022; Murray et al., 2007; Nahmani & Erisir, 2005), subplate cells (*NR4A2, DKK1, HCRTR2, MESP1, CHD1, CRYM, LMO3, ZFPM2, DRD1, KCNAB1, CTGF, BCL11B, TBR1, IGFBP5, NPR3, LMO7, NHLH2, TRPM3, TMEM178A, TLE4, WNT7B, NR4A3, SATB2*) (Miller et al., 2014; Nowakowski et al., 2017; Ozair et al., 2018), and radial glia (*PAX6, HES1, HES5, VIM, SLC1A3 [GLAST], NR2E1, TCF7L2, OTX1, GFAP, OLIG1, OLIG2, OTX2, SOX2, HOPX, NRG1, FAM107A, FBXO32, RBFOX1, PON2, CLU, TFAP2C, DDAH1* (Malatesta et al., 2008; Nowakowski et al., 2017; Pokhilko et al., 2021; Ramos et al., 2022)).

The LIANA (ligand–receptor analysis framework) tool (Dimitrov et al., 2022) was used to define ligand:receptor pairs between the thalamic projection neurons and subplate and radial glial cells in the frontal cortex. LIANA ranks interactions based on robustness across multiple ligand–receptor inference methods, producing a consensus interaction list across all single‐cell populations. This was filtered to identify cases where thalamic projection neurons were the ligand source and subplate or radial glial cells were the receptor targets. Finally, intracellular ligand pairs were manually removed from further analysis, and putative pairs were plotted using ggplot.

## 3 RESULTS AND DISCUSSION

### 3.1 Experiment overview

A single, anatomically well‐preserved human 20 PCW brain was subject to a fine dissection from eight coronal slabs (Figure 1a). From these slices, 18 regions were dissected, prepared for, and analyzed using our established PTM proteomics pipeline (Figure 1b) (Bogetofte et al., 2023; Elmkvist et al., 2024; Kang et al., 2018). Individual protein (non‐modified; Table S1a) and modified peptide (phosphorylation and free cysteines; Table S1b–d, sialylated N‐linked glycosylation; Table S1e,f, acetylation; Table S1g) quantifications were first subjected to basic quality control plotting. As is standard with single plex TMT experiments, the rate of missing values was very low for both unmodified proteins and modified peptides (Figures S1a,c and S2a,c). The sample‐by‐sample distributions of protein and peptide intensities were stable (Figures S1b,d and S2b,d).

### 3.2 Non‐modified proteins

**FIGURE 2:**
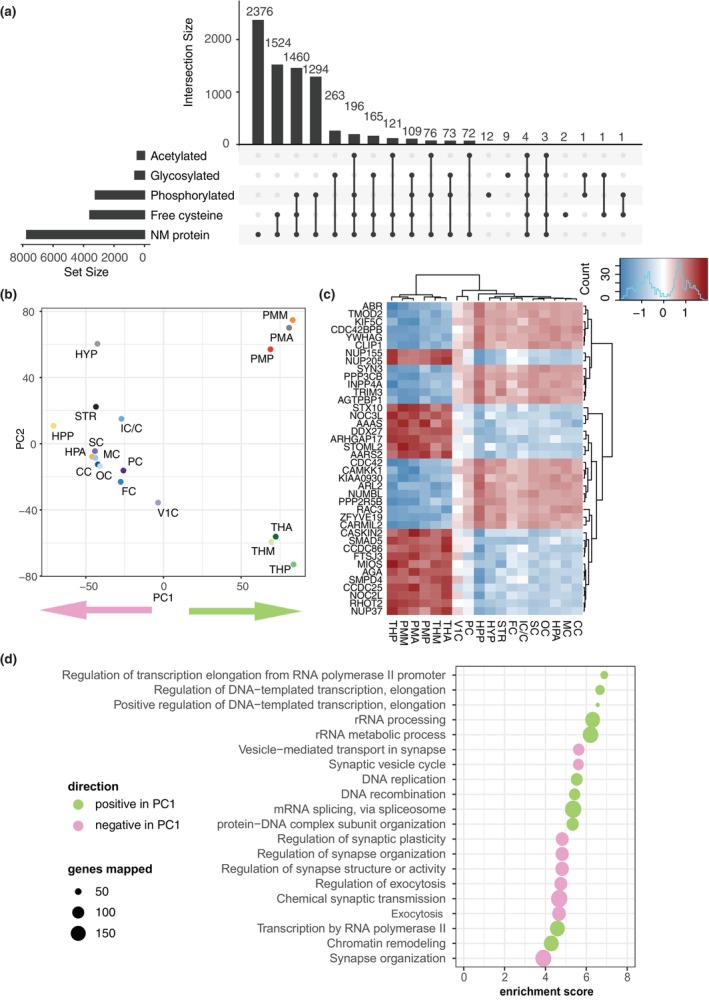
Samples from the pia mater and thalamus form distinct clusters separate from the other analysed regions. (a) Upset plot summarizing all peptides and proteins detected in the experiment. A total of 2376 proteins have no detected modifications, while at the opposite extreme, four peptides contain one of each modification. (b) PCA plot shows that PC1 separates pia mater and thalamus samples from all other regions, while PC2 separates the pia mater from the thalamus. (c) Heatmap showing the 20 proteins with the strongest loadings onto PC1 shows clear separation between pia mater and thalamus samples and all others. (d) Gene set enrichment analysis of all non‐modified proteins ranked by their PC1 loading shows enrichment of Biological Process terms for transcription in the pia mater and thalamus (green circles) and synaptic vesicle‐related terms in the other brain regions (pink circles).

A total quantity of 7736 unique non‐modified proteins were detected and quantified. A merged data table was created that combined information on multiple‐modified peptides (Table S2) and was used for downstream analysis. A total of 2376 proteins were only detected in the unmodified, protein‐level analysis. Free cysteines and phosphorylation were the most commonly detected PTMs, with four peptides carrying one of each modification type. Only 26 modified peptides came from proteins not also detected as non‐modified (Figure 2a). The mean number of modified peptides detected per protein for phosphorylation was 3.9, followed by 3.1 for glycosylation, 2.11 for free cysteines, and 2.06 for acetylation. Two hundred and thirty‐eight proteins had 10 or more phosphorylated peptides identified, with 37, 26, and 11 proteins having 10 or more free cysteine, glycosylated, and acetylated peptides identified respectively (Figure S3a). Pairwise correlations of modified peptide abundance (expressed as a proportion of unmodified protein abundance) show that in general, glycosylated peptides from the same protein are more strongly correlated with each other than phosphorylated peptides (Figure S3b). For proteins with both phosphorylated and glycosylated peptides, glycosylated peptides are more strongly correlated with other glycosylated peptides than with phosphorylated peptides (Figure S3c).

A PCA plot of non‐modified proteins showed clear clustering of samples according to their region of origin in Principal Component 1 (PC1) and PC2. PC1 separates the pia mater (PMA, PMM, PMP) and the thalamus (THA, THM, THP) samples from the other regions, while PC2 separates the pia and thalamus from each other, with the other regions occupying an intermediate position (Figure 2b). Proteins driving separation of the pia and thalamus from other regions in PC1 include SMAD, CCDC, and NUP protein family members (Figure 2c). Gene set enrichment analysis of all proteins ranked by their PC1 loading value shows that the pia and thalamus are enriched for biological processes including RNA transcription, rRNA processing, and DNA recombination and replication, suggesting these regions are undergoing substantial active cell division at this developmental stage (Figure 2d). The samples from the other regions, which include the cortex and hippocampus, are enriched for proteins involved in vesicle cycling and synaptic plasticity and organization (Figure 2d). These enrichments are driven by proteins including CAMKK1, SYN3, and YWHAG (Figure 2c).

### 3.3 Cortical transcription factors

**FIGURE 3:**
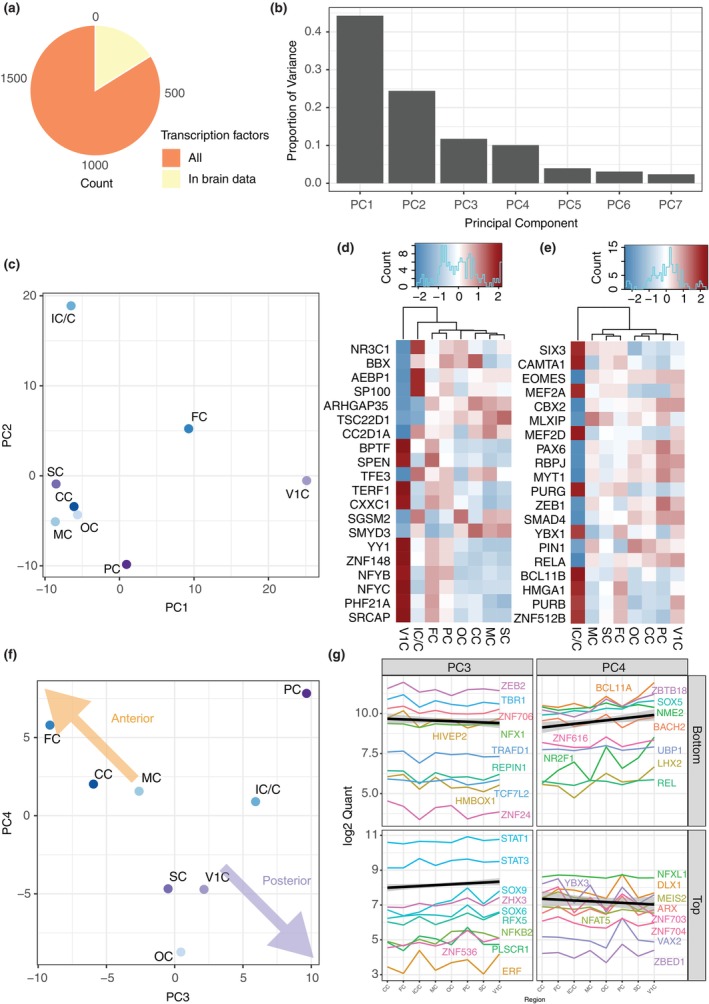
Some transcription factors in the cortex may show an anterior posterior gradient. (a) Pie chart showing that 332 transcription factors from a master list of 1639 were detected in our experiment. (b) Bar plot showing the proportion of variance explained by each of the first seven principal components. (c) PCA plot of components 1 and 2 clearly separates insular cortex/claustrum, frontal cortex and primary visual cortex from each other and from all other samples. (d) Heatmap shows the 10 proteins at the tails of loadings on PC1, which clearly separates primary visual cortex from other regions. (e) Heatmap shows the 10 proteins at the tails of loadings on PC2, which clearly separates insular cortex/claustrum from other regions. (f) PCA plot of components 3 and 4 shows rough anterior–posterior organization of cortical regions (g) Line plots show transcription factors driving PC3 and PC4 and their abundance across cortical regions. The thick black line shows the average trend from anterior to posterior of these transcription factors, with a gray shadow showing the 95% confidence interval.

It has been well described that gradients of transcription factors (TFs) determine cell fate and regional localization at earlier stages of neural development (Johnson et al., 2009; Sansom & Livesey, 2009), but less focus has been given to later stages. For this reason, we intersected our non‐modified protein list with a list of 1639 established TFs obtained from Lambert et al. (2018). Three hundred and thirty‐two of these TFs were detected in our dataset (Figure 3a). The most common PTM on these proteins was phosphorylation, with 183 of the transcription factors containing at least one phospho‐peptide. Only one transcription factor, AEBP1 contained a glycosylated peptide (Figure S4a). AEBP1 has multiple isoforms, one involved in extracellular matrix remodeling and secreted (Blackburn et al., 2018), with another associated with transcriptional repression (Kim et al., 2001).

Using only the cortical samples, we performed a PC analysis on the transcription factors, with PC1 (44.3% of total variance, Figure 3b) clearly separating Primary Visual Cortex (V1C) from the other cortical regions (Figure 3c). V1C is enriched for TFs involved in lysine methylation (CXXC1, NFYB, NFYC), and SWI SNF superfamily TFs involved in negative regulation of transcription (YY1, SRCAP, BPTF, Figure 3d). Insular cortex/claustrum was the outlying sample in PC2, driven by low levels of classical TFs involved in brain development such as EOMES, SMAD4, and PAX6 (Figure 3e). This may be because the insula does not have a directly underlying ventricular zone or subventricular zone.

Plotting of PC3 and PC4, which account for 21.7% of the total variance in transcription factor abundance, shows anterior to posterior ordering of regions, with the parietal cortex as an exception (Figure 3f). The transcription factors driving these components, which include STAT1 and 3, SOX5, 6 and 9 and LHX2, show subtle trends in abundance across regions from anterior to posterior, suggesting transcription factor gradients may still play a role in defining regional identity at this stage of development (Figure 3g).

### 3.4 RNA–protein correlations

**FIGURE 4:**
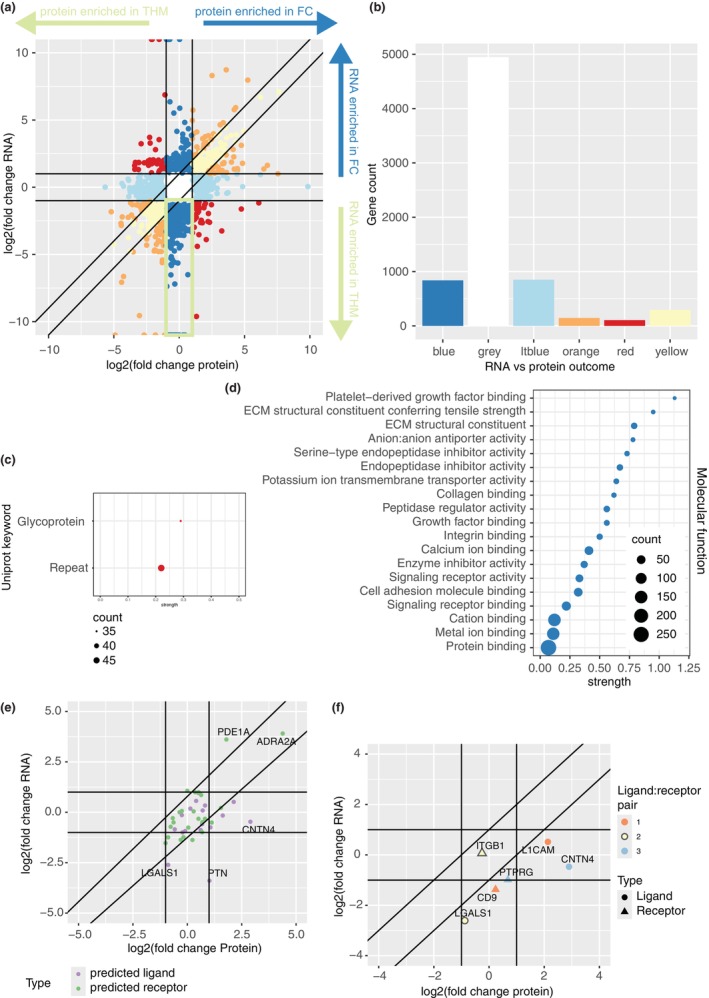
Comparison of RNA and protein abundance in matched regions highlights possible ligand:receptors pairs between the thalamus and frontal cortex. (a) Dot plot shows RNA and protein abundance differences between the medial thalamus and frontal cortex regions. Points are colored on the basis of their agreement or disagreement between the RNA and protein measurements. Yellow dots show proteins where the protein variability between regions is consistent with the RNA variability. Orange proteins show enrichment in the same region as the RNA, but with disagreement to the magnitude of enrichment. Light gray proteins are equally abundant in both regions. Blue proteins show an enrichment in a region by only RNA (dark blue) or only protein (light blue). Red proteins disagree, showing enrichment in different directions for RNA versus protein. (b) Bar plot shows that most proteins are light gray, in that the RNA and protein agree they are not enriched in either brain region. Over 700 proteins fall into each of the blue categories, showing that RNA and protein measures do not always agree. (c) Gene ontology enrichment analysis of red class proteins shows an enrichment for the Uniprot keyword glycoprotein, which tend to be cell surface or secreted proteins. (d) Gene ontology enrichment for proteins where the RNA is enriched in the thalamus, but the protein is present at equivalent levels in both thalamus and frontal cortex, includes Molecular Function terms for extracellular facing and extracellular matrix categories. (e) Scatterplot showing the location on Figure A of potential ligand:receptor pairs highlighted by LIANA analysis. (f) Scatterplot highlight three protein pairs that inhabit the appropriate section of the plot to be considered higher confidence ligand:receptor pairs.

We have previously shown that discrepancies in fold change between regions at the RNA and protein level can highlight interesting categories of protein, such as secreted proteins, that may be used to signal between regions (Carlyle et al., 2017). To compare RNA differences between regions with protein, we obtained a normalized RNA count matrix from the BrainSpan project from PCW 19 (Li et al., 2018). BrainSpan regions were matched to our dissected regions, and RNA counts were added to the master table (Table S2). As it is well established that two regions, the thalamus and PFC, are anatomically distant but interconnected by axonal projections that carry secreted proteins (Sato et al., 2022), RNA and protein fold changes were compared between the medial thalamus and prefrontal cortex samples (Figure 4a). Most genes showed no substantial difference in abundance between the two regions in either RNA or protein (4949 proteins, Figure 4b). Two hundred and ninety‐five genes (yellow) show comparable magnitudes of fold changes in both protein and RNA between thalamus and PFC. Of these, 146 genes' fold changes were in the same direction (either increase or decrease, respectively), but of a different order of magnitude. On the other hand, 106 of these genes show an opposing direction of fold change in RNA and protein, and are shown in red (Figure 4b). Gene ontology (GO) term enrichment analysis shows that these latter proteins are enriched for the Uniprot keyword glycoprotein, suggesting they are secreted or extracellular facing (Figure 4c).

### 3.5 Ligand:Receptor interactions

We reasoned that thalamic‐specific secreted proteins would show RNA expression primarily in the thalamus, while protein would be present in both the thalamus and the cortical area to which it projects its axons. This subset of proteins is indicated by a green box in Figure 4a. GO enrichment analysis shows that secreted and extracellular membrane proteins are found in this portion of the plot (Figure 4d). We therefore used the LIgand–receptor ANalysis framework (*LIANA* [Dimitrov et al., 2022]) and CellPhoneDB (Efremova et al., 2020; Vento‐Tormo et al., 2018) tools to identify possible high confidence ligand–receptor pairs in a single cell RNAseq embryonic human dataset (Bhaduri et al., 2021) at 20 PCW, and plotted the location of these putative pairs on our RNA–protein fold change plot (Figure 4e). Only two putative ligands showed the expected distribution, with a further three showing no region enrichment for RNA, but strong enrichment for protein in the frontal cortex. Known intracellular interactors were removed from analysis, leaving three high confidence ligand:receptor pairs (Figure 4f). LGALS1 RNA is enriched in the thalamus, with protein present at equivalent levels in both regions. It is predicted to bind to the ITGB1 receptor, found at equivalent levels of RNA and protein in both regions. L1CAM and CNTN4 ligands' RNA expression was similar in both regions, but protein levels were higher in the cortex. Both these ligands were heavily glycosylated, with 21 and 16 glycosylated peptides identified from each protein, respectively (Table S2). Across all putative pairs, receptors generally showed equivalent levels of RNA and protein in both regions (Figure 4e). Both ITGB1 and PTPRG were glycosylated, with 9 and 2 peptides identified, respectively (Table S2).

### 3.6 Phosphorylation

**FIGURE 5:**
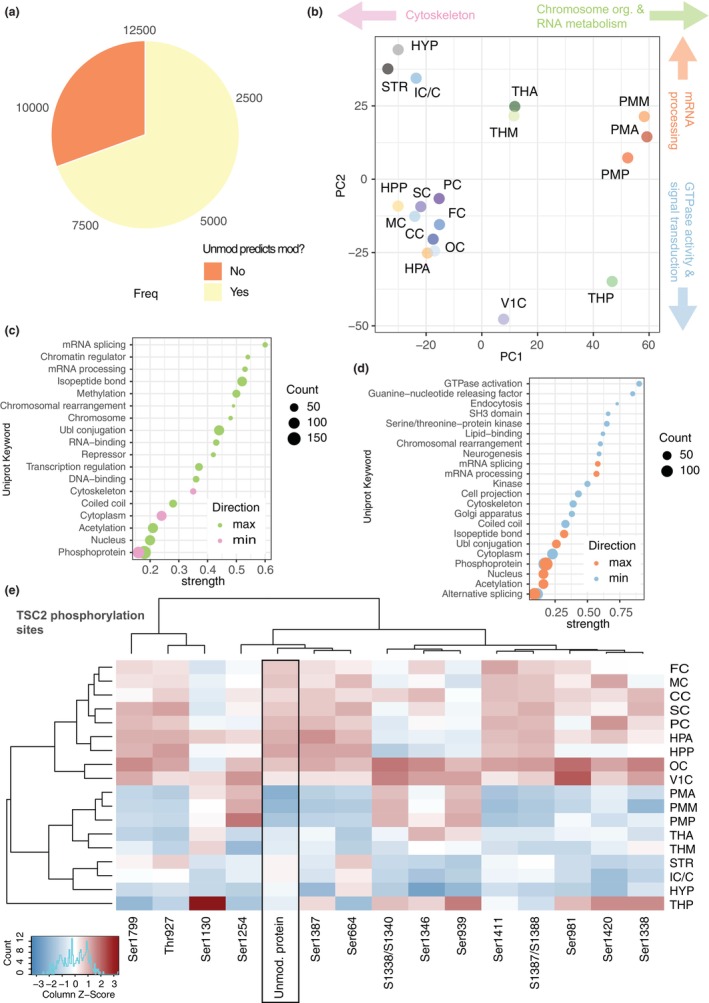
The phosphorylated peptidome is significantly different in the pia mater compared to other regions, with the thalamus as an intermediate cluster. (a) A linear model predicting abundance of phosphorylated peptide by abundance of non‐modified protein is significant for 8781 of the 12,641 detected phosphorylated peptides. (b) PCA outcome after PC analysis of only the proteins for which a linear model did not significant explain phosphorylated peptide abundance. Pia mater is once again clearly separated from other regions, but posterior thalamus is distinct from the other two thalamic samples. (c) GO analysis shows enrichment in PC1 tails for Uniprot Keywords including RNA processing and transcription regulation in the pia mater direction, while cytoskeletal terms are enriched in the opposite direction. (d) GO analysis shows enrichment in the PC2 tails for Uniprot Keywords related to vesicle function and signal transduction in the primary visual cortex direction, and mRNA processing in the hypothalamus direction. (e) Heatmap shows abundance of phosphorylated peptides in TSC2, compared to levels of unmodified protein. Posterior thalamus shows relatively high levels at multiple phosphorylation sites on TSC2, despite having lower levels of unmodified protein.

Moving to PTMs, 3226 unique proteins were found to be phosphorylated on 12,641 unique peptides. Different combinations of phosphorylated residues were detected in 1960 of these unique peptides (Table S2). For the vast majority of phosphorylated peptides (8781), a linear model with non‐modified protein abundance as the explanatory variable significantly predicted phosphorylated peptide abundance, suggesting phosphorylation of the peptide is strongly related to protein expression (Figure 5a). Density plots of the residuals from this linear model show that the distribution of residuals in subcortical regions is wider than in cortex and hippocampus (Figure S4b), implying that modification is less predictable by non‐modified protein abundance in these regions. The abundance of phosphorylated peptides in the posterior hippocampus, motor cortex, and somatosensory cortex is most strongly predicted by non‐modified protein abundance. We performed PCA on the 3860 remaining peptides that may be more dynamically modified. As we saw with non‐modified proteins, PC1 mostly separates the Pia Mater from other regions, with PC2 adding some interesting clustering of regions. The posterior thalamus was separated in both PCs from the anterior and medial thalamus (Figure 5b). GO enrichment analysis was performed on the 5% of proteins occupying each tail of the PC loadings. PC1 had enrichment for terms related to chromosome organization and RNA metabolism (Figure 5c), consistent with regulation of cell division and/or epigenetic regulation of transcription at higher levels in the pia mater and posterior thalamus at this developmental stage. In the opposite direction on PC1, there was enrichment for cytoskeletal proteins. GTPase activity and cell projections are enriched toward the lower values of PC2 (Figure 5d), which separates the primary visual cortex from other cortical regions, and posterior thalamus from the other thalamic regions. In the future, it would be interesting to assess whether phosphorylation in certain organelles, such as the nucleus, may be more stable over different postmortem intervals than those in other subcellular locations.

We then focused on the phosphorylation sites quantified in TSC2 (Figure 5e), a protein driving the “GTPase activation” GO category. Mutations in TSC2 protein can cause severe neurodevelopmental problems that may result in intellectual disability and autism spectrum disorder (Huang & Manning, 2008). TSC2 forms a complex with TSC1 which interacts with the mTOR signaling pathway to regulate cell growth and division in response to cellular energy needs (Bassetti et al., 2021). In our study, we detected phosphorylation at 15 different sites in TSC2. All but one (pSer1254) of these sites have been previously identified (Ochoa et al., 2020). Ser939 is phosphorylated by AKT via the PI3K pathway (Manning et al., 2002), which plays a significant role in coordination and integration of growth factor signaling, axon guidance cues, and neuronal migration (Sharma & Mehan, 2021). Modification at this Ser939 site was not strongly related to non‐modified protein abundance, with the posterior thalamus having the highest level of phospho‐peptide, despite a low level of non‐modified protein (Figure 5e).

### 3.7 Sialylated glycosylated peptides

**FIGURE 6:**
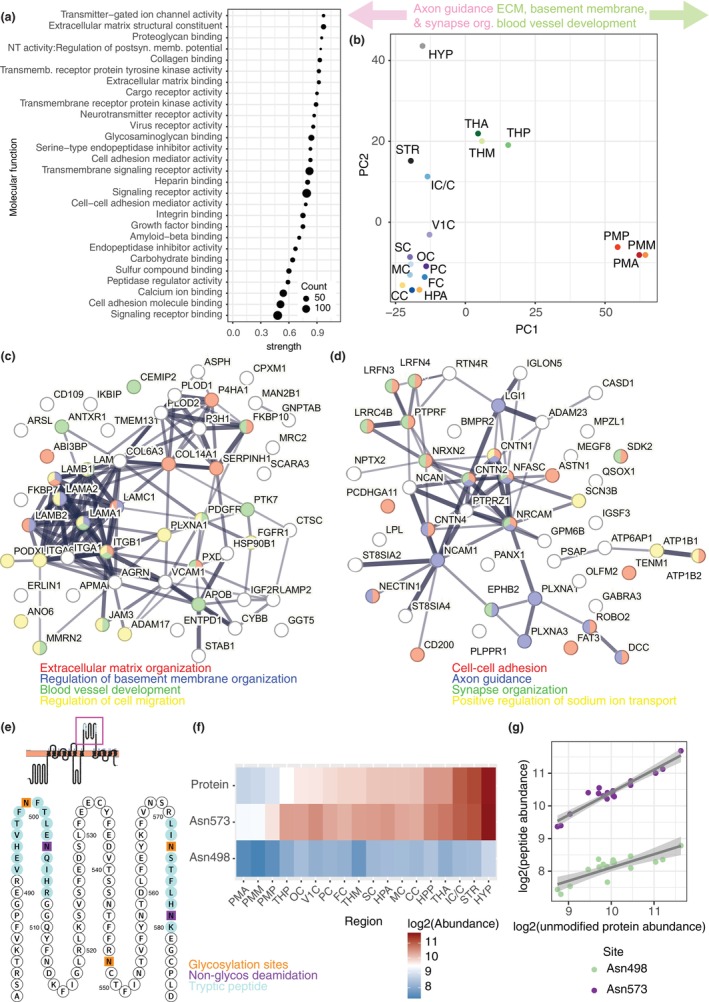
Glycosylated peptides are involved in extracellular matrix and basement membrane organization in the pia mater, and synaptic organization in cortical regions. (a) GO enrichment analysis of all sialylated N‐linked glycosylated proteins shows enrichment for expected GO Molecular function terms. (b) Pia mater samples are strongly separated from all other samples in a PCA plot of components 1 and 2. (c) String network plot shows proteins in PC1 tails that are enriched in pia mater. Nodes are colored by enriched GO terms; red—extracellular matrix organization, blue—Regulation of basement membrane organization, green—blood vessel development, and yellow—regulation of cell migration. (d) String network plot shows proteins in PC1 tails that are enriched in cortical regions. Nodes are colored by enriched GO terms; red—cell–cell adhesion, blue—axon guidance, green—synapse organization, and yellow—positive regulation of sodium ion transport. (e) Protter diagram shows the extracellular loop of SV2A protein. Tryptic peptides quantified in this study are shown in light blue, with known glycosylation sites in orange. Purple shows sites where we detected non‐glycosylation related deamidation. (f) Heatmap shows that glycosylation at both sites is strongly related to non‐modified protein abundance, though acetylation at Asn 498 is detected at a lower abundance than Asn 573. (g) Scatterplot shows the strong relationship between non‐modified protein abundance and glycosylated peptide levels.

A total of 1924 unique sialylated N‐linked glycosylated peptides were detected from 629 unique proteins. Non‐modified protein abundance was predictive of glycosylation for the majority of peptides (1710 out of 1924). Glycosylation had the highest rate of prediction for any of the PTMs (Figure S5) suggesting that glycosylation is the least dynamically modified PTM. As expected, GO enrichment analysis of all glycosylated proteins showed an enrichment for secreted, membrane resident (extracellular facing), and extracellular matrix proteins (Figure 6a). PCA showed separation of the three pia samples from the others in PC1, whereas PC2 separated the thalamus, hypothalamus, striatum, and insular cortex/claustrum from the other regions (Figure 6b). A GO enrichment analysis on the 5% of proteins in each tail of the PC1 loadings showed an enrichment for extracellular matrix and basement membrane organization and blood vessel development in the pia samples (Figure 6c), while all other regions were enriched for axon guidance and neurodevelopment‐related process GO terms (Figure 6d). To focus on a specific protein, SV2A is a membrane transporter involved in synaptic function that has three established glycosylation sites, two of which we quantified in this study; at Asparagine 498 and Asparagine 573 (Figure 6e). In combination, glycosylation at these sites has been shown to be required for appropriate sorting of SV2A to the synapse (Kwon & Chapman, 2012). The abundance of both glycosylation sites was strongly correlated with protein abundance across brain regions, with THP, OC, and V1C exhibiting slightly higher levels of glycosylation than would be predicted by total protein levels. Asn498 glycosylation, on the other hand, was detected at lower abundance and with a lower degree of correlation (Figure 6e,f).

### 3.8 Lysine acetylated peptides

**FIGURE 7:**
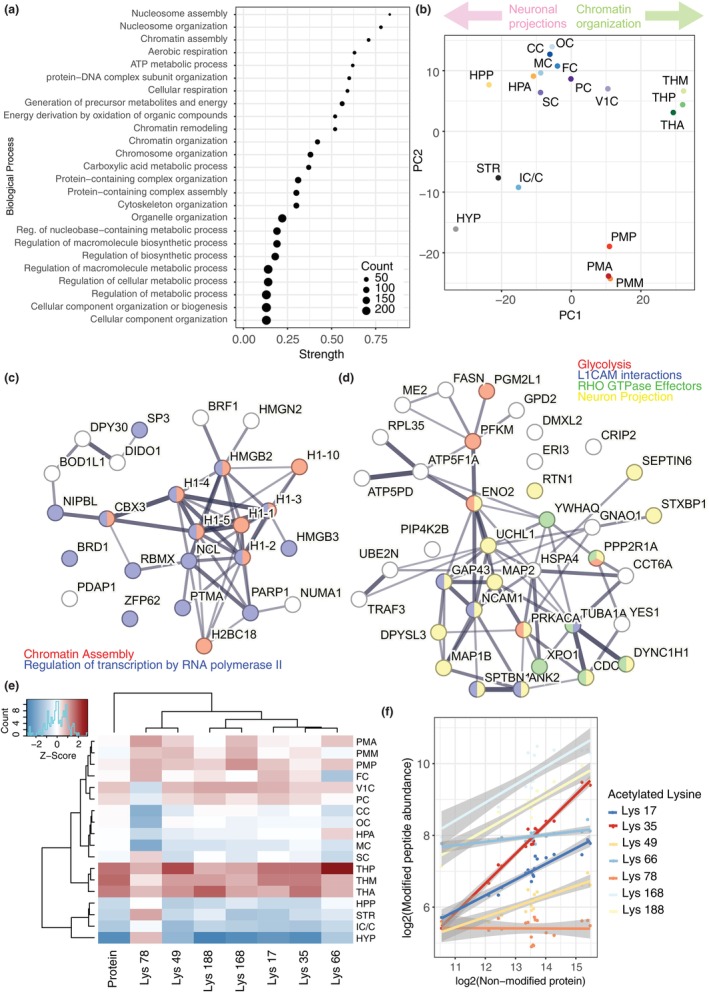
Acetylated peptides are involved in transcription regulation in the thalamus, and neuronal differentiation in other regions including the hypothalamus, striatum, and posterior hippocampus. (a) GO enrichment analysis of all acetylated proteins shows enrichments for expected biological process terms. (b) PCA plot shows clear clustering of thalamus samples from others in PC1 and pia mater samples from others in PC2. (c) String network plot shows proteins in PC1 tails that are enriched in thalamus. Nodes are colored by enriched GO terms; red—chromatin assembly and blue—regulation of transcription by RNA polymerase II. (d) String network plot shows proteins in PC1 tails that are enriched in the opposite direction to thalamus. Nodes are colored by enriched GO terms; red—glycolysis, blue—L1CAM interactions, green—RHOS GTPase effectors, and yellow—neuron projection. (e) Heatmap shows abundance of acetylated peptides in the linked histone HIST1H1B. Lys 78 abundance has no relationship to the abundance of non‐modified protein. (f) Scatterplot comparing non‐modified protein levels to acetylated peptide levels. Some sites, such as Lys 35, can be strongly predicted by protein level, whereas others show a weaker or no association.

Nine hundred and sixty‐five unique lysine acetylated peptides were detected from 472 proteins. Acetylation represented the least predictable modification based on protein levels (Figure S5), suggesting this may be the most dynamic PTM. GO enrichment analysis of all acetylated proteins highlighted proteins involved in metabolic processes and nucleosome organization, as expected for this category of PTM (Figure 7a). PCA showed 4 distinct groupings in the first two principal components; all three pia samples were grouped, as were all three thalamus samples, with striatum, insular cortex/claustrum, and hypothalamus separated from the other cortical and hippocampal samples (Figure 7b). GO enrichment analysis on the 5% of proteins occupying the PC loading tails showed an enrichment for terms including chromatin assembly and regulation of transcription by RNA polymerase II to the right of the PCA plot (Figure 7c) that included the pia and thalamic clusters. Neuron projection and L1CAM interactions were enriched to the left, including in the hypothalamus, striatum, and hippocampus (Figure 7d). The latter group was also enriched for the glycolysis GO term, which is relevant since a transition of metabolic state from aerobic glycolysis to oxidative phosphorylation is a key feature of neuronal differentiation (Zheng et al., 2016).

Histone acetylation is a dynamic set of modifications with significant downstream effects on transcription. In our dataset, we detected seven acetylated lysines on HIST1H1B, a linker histone essential for higher order structure of nucleosomes and transcriptional control (Behrends & Engmann, 2020) (Figure 7e). While some sites were very strongly correlated with protein abundance, such as lysine 35 acetylation, others were more variable. Acetylation at lysine 78 was unrelated to protein abundance (Figure 7f), with high levels of modified peptide in the striatum and hypothalamus despite low levels of protein (Figure 7e).

## 4 CONCLUSION

In this study, we presented a new dataset containing information on protein and PTM peptide abundance across 18 brain regions in a single, anatomically well‐preserved postmortem human brain. We hope this data will function as a useful reference for investigators working on mid‐fetal human brain development, while inspiring confidence in the robustness of the analytical techniques, and the types of questions that could be answered using them. Given this data are from a single brain and the resolution of the dissection was low (e.g., not providing resolution for different thalamic nuclei), we aimed to highlight the types of approaches that can be used with this data, without making firm statements about novel biological knowledge that would require a greater sample size. Improving the spatial resolution of dissections for these experiments will increase the utility of the information we obtain.

The primary limitation of this work is the sample size of 1. While the sample did not show major brain abnormalities nor common genetic defects, there may be other underlying problems not acknowledged on this general screen. The complexity of the human brain, alongside the interindividual variability characteristic of this type of specimen, suggests caution should be applied when interpreting findings from data derived from one donor only.

With regards to the posttranslational aspects of the work, there are two main potential confounds: the postmortem interval and our understanding of how PTMs may change observability of peptide species. Previous studies have shown that the postmortem interval is associated with changes in abundance of some proteins and phosphorylated sites, while many remain stable (Carlyle et al., 2021; Grubisha et al., 2021) as the interval increases. Other PTMs have been less investigated. Therefore, increasing the sample size is essential in order to fully understand the relationship between PTMs, postmortem interval and human brain development and disease. PTMs continue to be of strong interest to the community, as multiple lines of evidence implicate kinase and phosphatase enzymes in diseases such as schizophrenia (Grubisha et al., 2021).

Finally, the complexity of the association between non‐modified protein and modified peptides is not yet fully understood, and there is no clear consensus as to how to express this relationship. It is possible that PTM modification at one residue can alter the observability of peptides by MS, which makes assessment of the extent of modification more complex than the ratiometric approach commonly used in immunoblotting and molecular biology. In early stages of our analysis here, it was clear that the abundance of non‐modified protein frequently had a stronger effect than the abundance of modified peptide when expressing modification as a proportion, as this value was generally higher and more variable than the levels of modified peptide. For this reason, the data presented here usually show the quantifications from both non‐modified protein and modified peptide so that the relationship between them can be better understood.

The goal is that this data can support ongoing research and spark new lines of investigation, while increasing enthusiasm for future studies of this type. As MS technology has recently leapt forwards at breath‐taking pace, with significant increases in resolution, accessible proteome depth, and reproducibility (Messner et al., 2023), now is the time to broaden our knowledge of the 'omic domain that lies closest to biological function.

## AUTHOR CONTRIBUTIONS

**S. Bandiera:** Experiment design, sample preparation, data analysis, manuscript writing and review. **H. Bogetofte:** Experiment design, sample preparation, data acquisition, data analysis, manuscript writing and review. **P. Jensen:** Sample preparation, data acquisition, data analysis, and manuscript review. **R. Hussain:** Data analysis, manuscript writing, and manuscript review. **S. A. Nischal:** Data analysis and manuscript review. **L. Rihova:** Manuscript writing. **G. J. Clowry:** Sample acquisition, sample preparation, manuscript review, and funding. **M. R. Larsen:** Experiment design, sample preparation, data acquisition, data analysis, manuscript review, and funding. **Z. Molnár:** Experiment design, sample preparation, manuscript review, and funding. **B. C. Carlyle:** Experiment design, sample preparation, data analysis, manuscript writing and review, and funding.

## FUNDING INFORMATION

Tissue was provided from the Human Developmental Biology Resource funded by grant Wellcome Trust/MRC 099175/Z/12/Z/MR/R006237/1 to G. J. Clowry. B. C. Carlyle is supported by an Alzheimer's Research UK Senior Research Fellowship (ARUK‐SRF2022A‐012) and a BD^2^ Discovery Grant (DG240508). Z. Molnár's laboratory was supported by the following funding sources: BBSRC Project Grant (BB/X008711/1), MRC Project Grant Molnár (PI) and Mann (Co‐PI) (MR/W029073/1), Einstein Stiftung Berlin with Prof Britta Eickholt at Charité‐Universitätsmedizin Berlin, Germany as part of Z. Molnár being Einstein Fellow at Charité Universitätsmedizin Berlin (2020–2026), and an Oxford Martin School Grant (Bayley, Szele, Molnár). M. R. Larsen laboratory is supported by the Lundbeck Foundation (grant number R336‐2020‐1113), the Villum Center for Bioanalytical Sciences at University of Southern Denmark, and the Danish Agency of Higher Education and Science infrastructure MS platform PLATO (grant number 5229‐00012B). S. Bandiera was supported by MRC Graduate studentship. H. Bogetofte was supported by the Lundbeck Foundation postdoc fellowship (R380‐2021‐1425).

## CONFLICT OF INTEREST STATEMENT

BCC receives funding from sponsored research agreements with GSK and Ono Pharmaceuticals for work not associated with this paper.

## Supporting information


Figure S1.

Figure S2.

Figure S3.

Figure S4.

Figure S5.

Table S1.

Table S2.

## Data Availability

Mass spectrometry raw files are available on PRIDE with reference PXD065788. Processed quant files are available as supplementary data to this manuscript. Code and input data are available on github at: https://github.com/Carlyle‐Lab/PCW20_PTM_Proteomics.
