# Supplementary figures and images for: A posttranslational proteomic survey of a single anatomically preserved human 20‐week postconception brain
Source: J Anat. 2026 May 3:10.1111/joa.70170. Online ahead of print. doi: 10.1111/joa.70170 (PMC13399142; doi:10.1111/joa.70170)

Figure S1

Non-modified protein

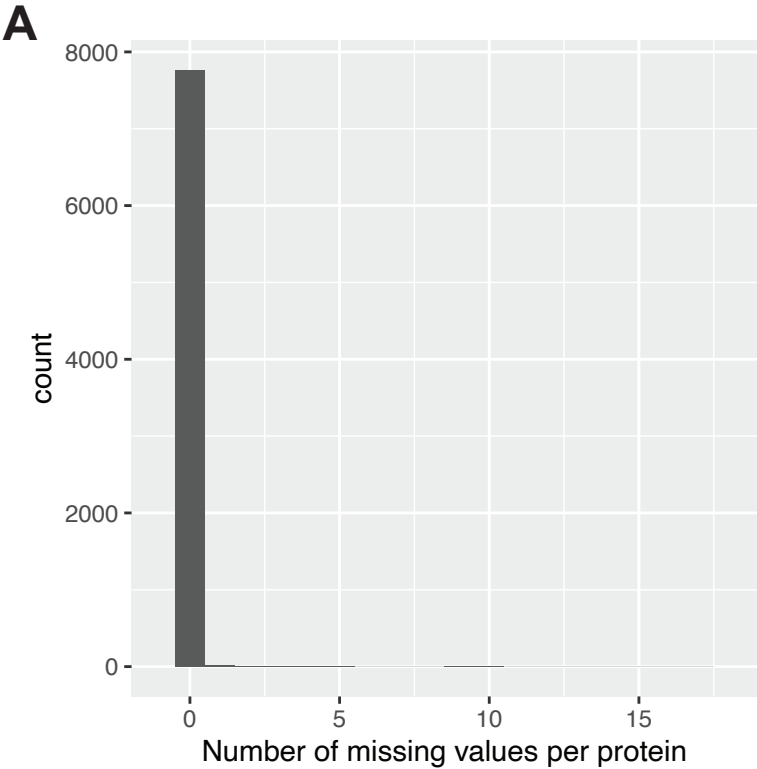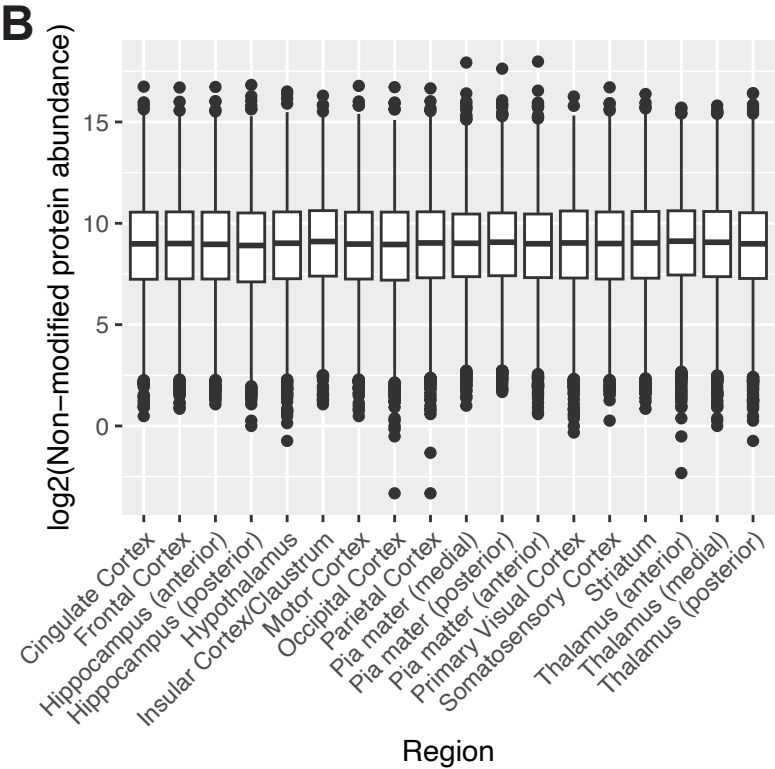

Phosphorylated and SIA (free cysteine) peptides

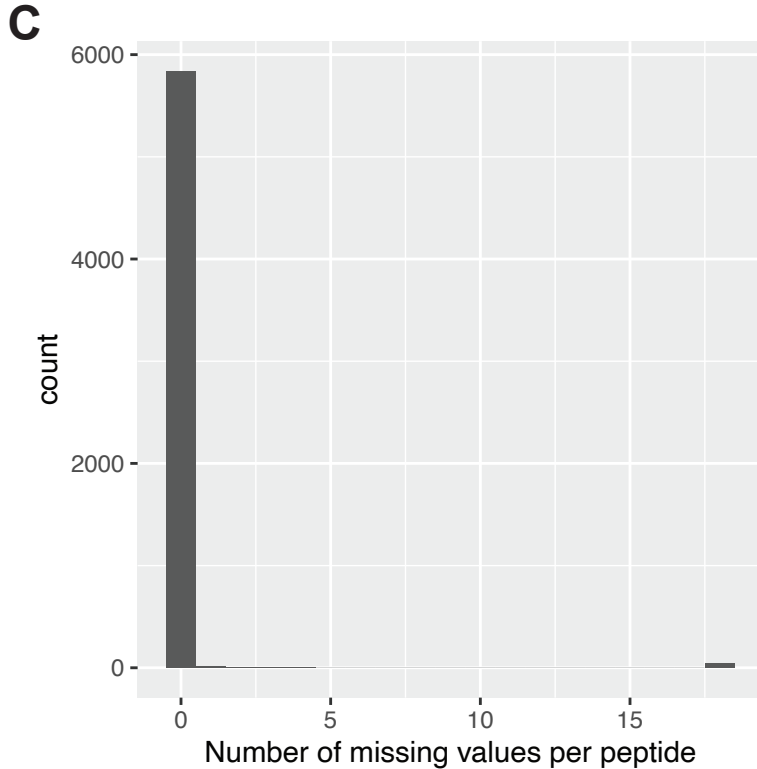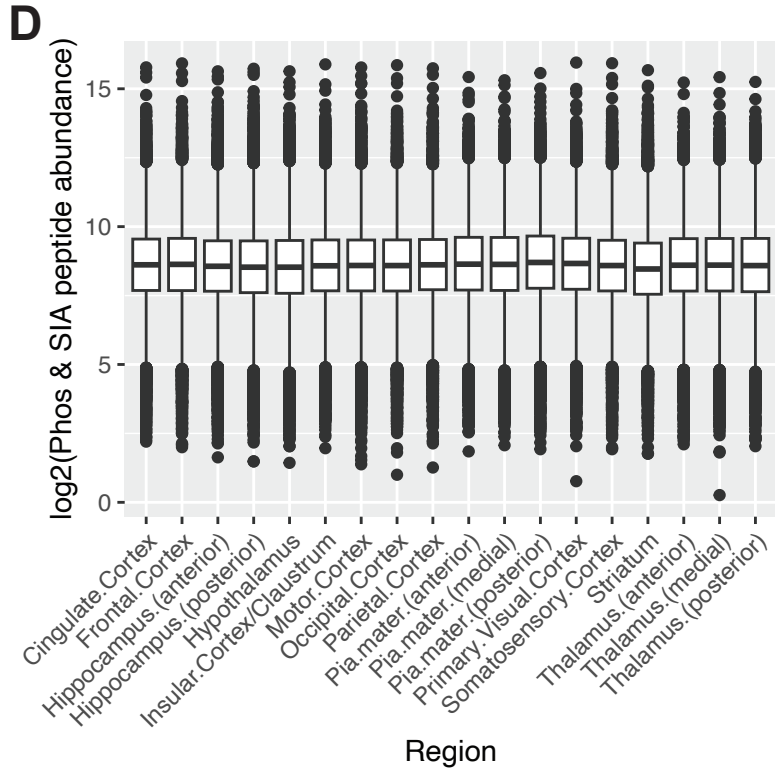

Supplement: Supplementary file 1 — Figure S1. [file JOA-9999-0-s002.pdf]

Figure S2

Sialylated N-linked Glycosylated peptides

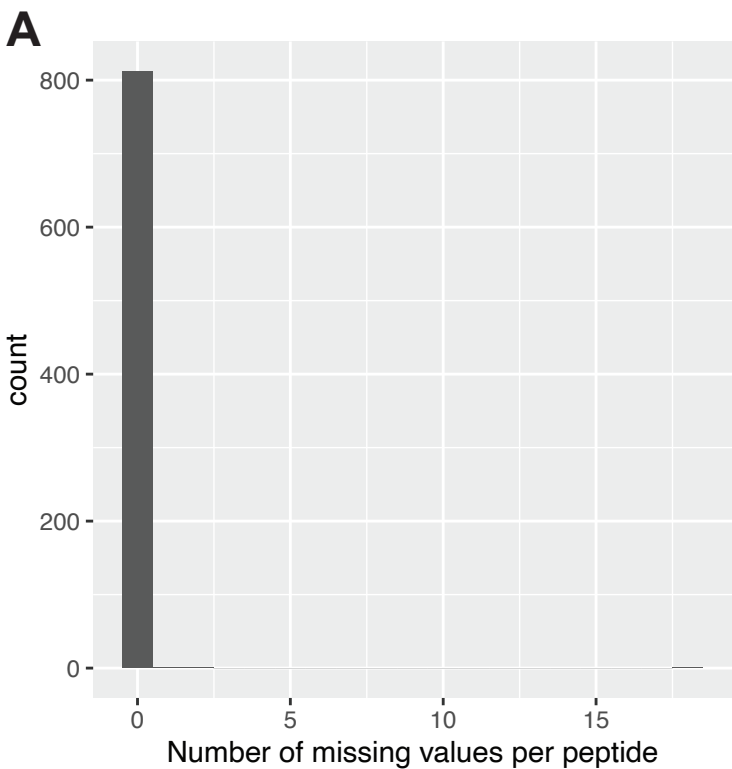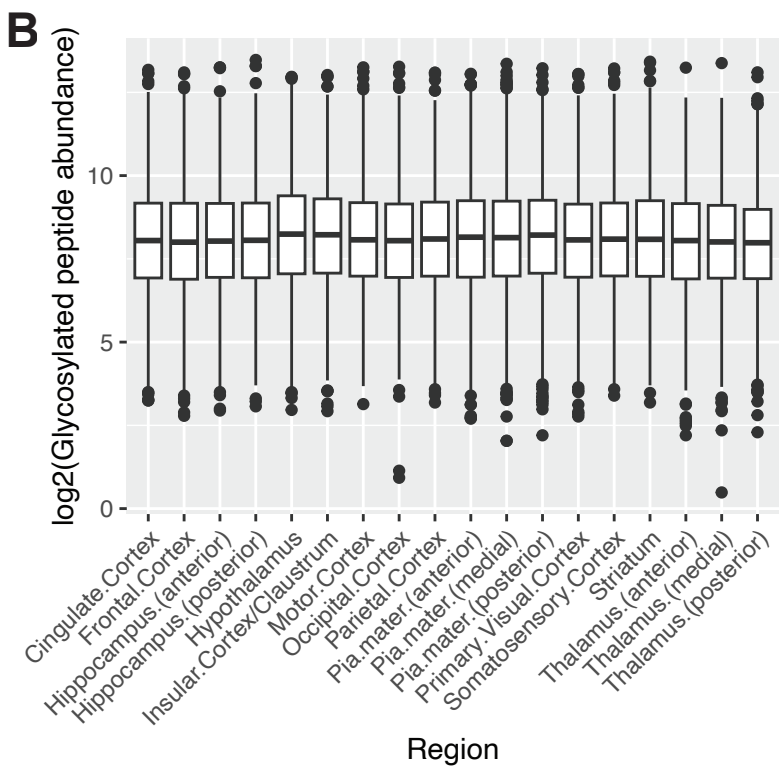

Acetylated peptides

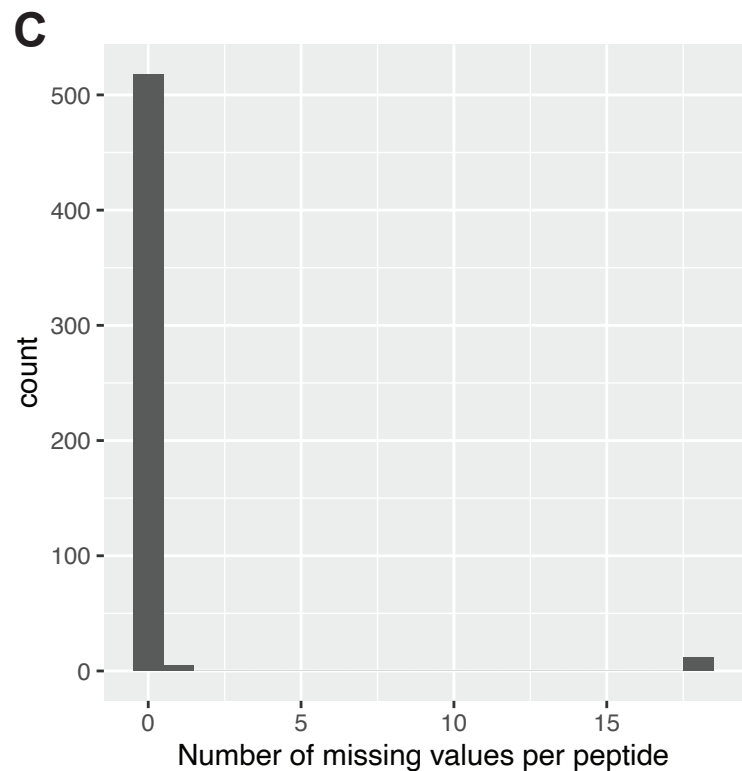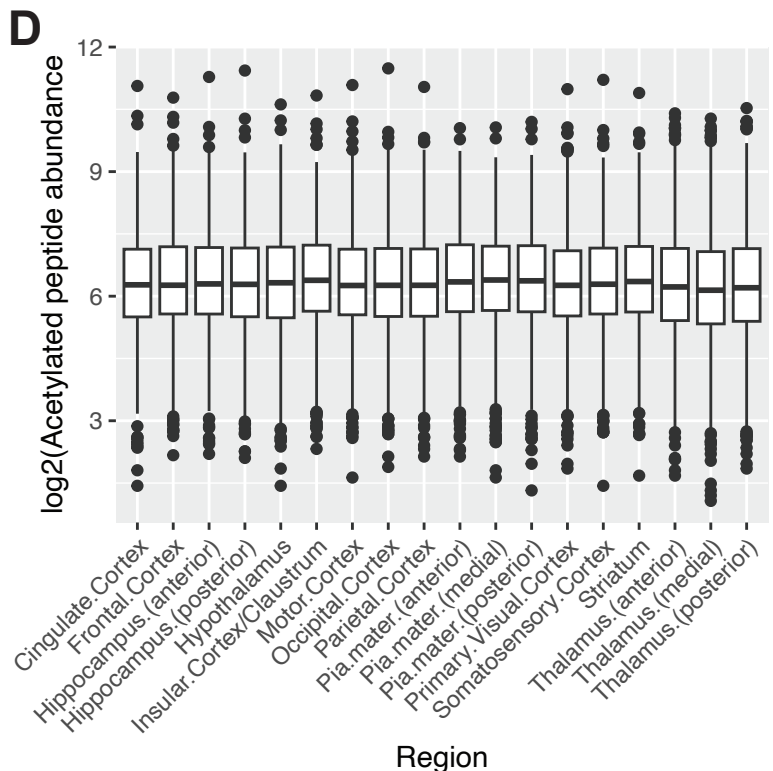

Supplement: Supplementary file 2 — Figure S2. [file JOA-9999-0-s007.pdf]

**Figure S3**

**A**

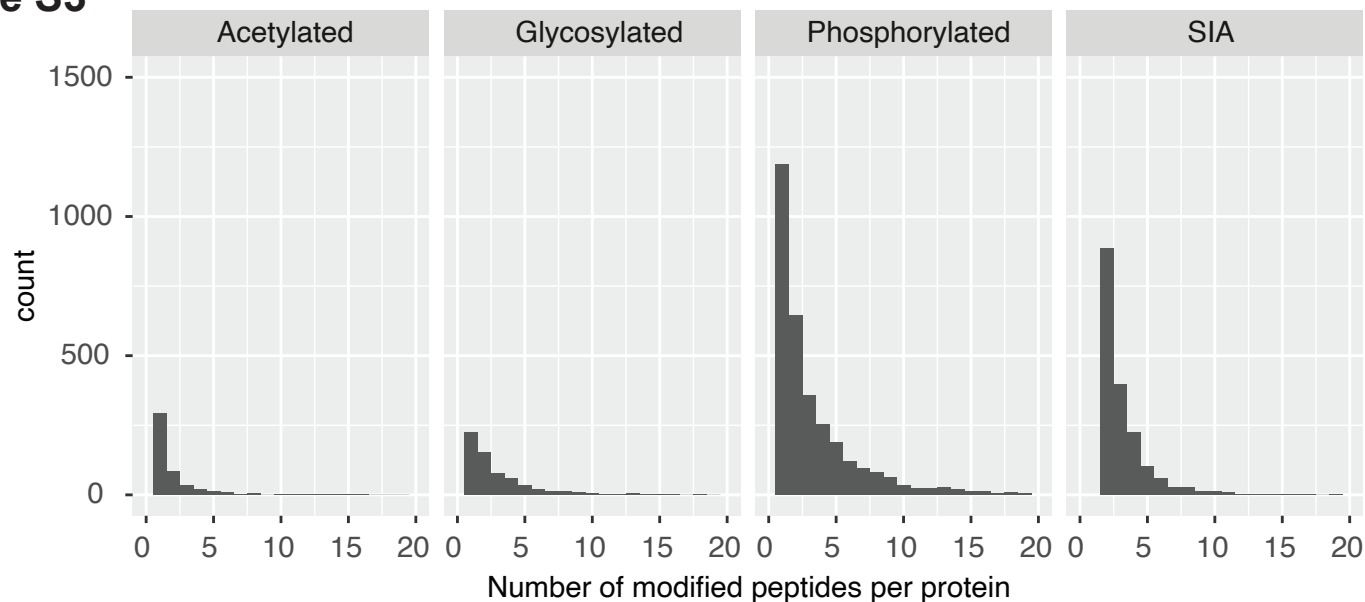

**B**

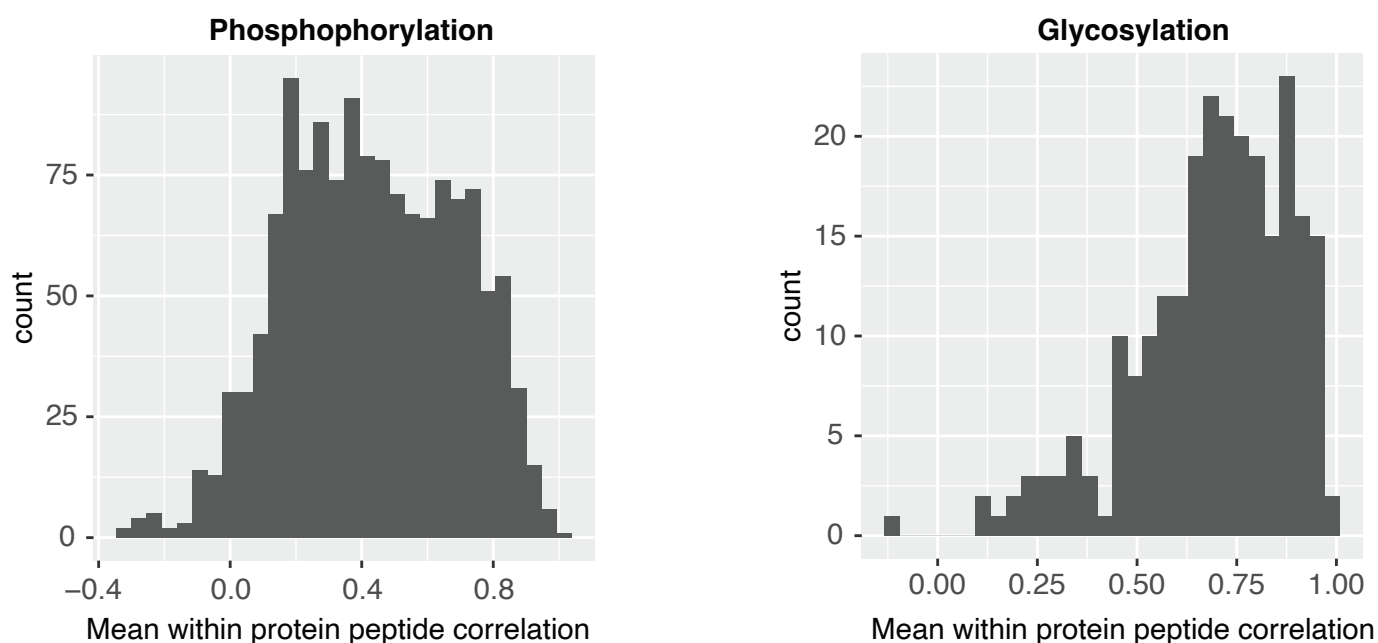

**C**

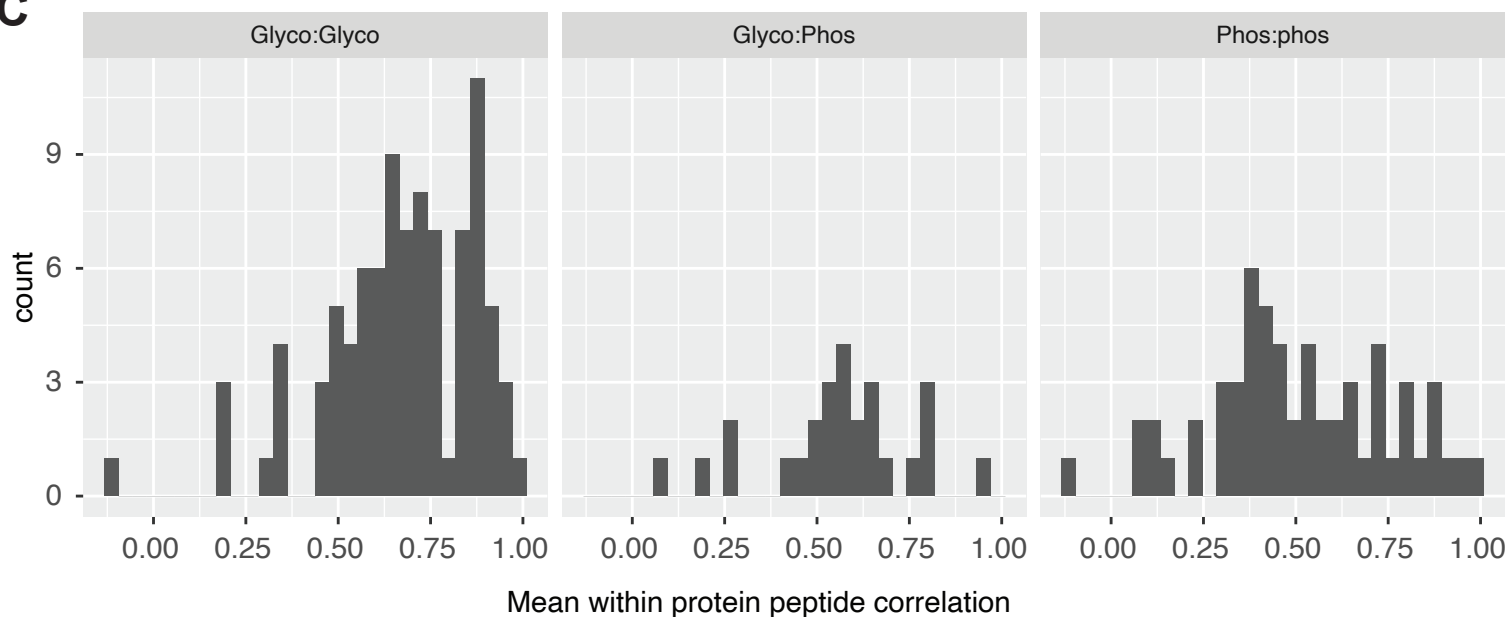

Supplement: Supplementary file 3 — Figure S3. [file JOA-9999-0-s003.pdf]

**Figure S4****A**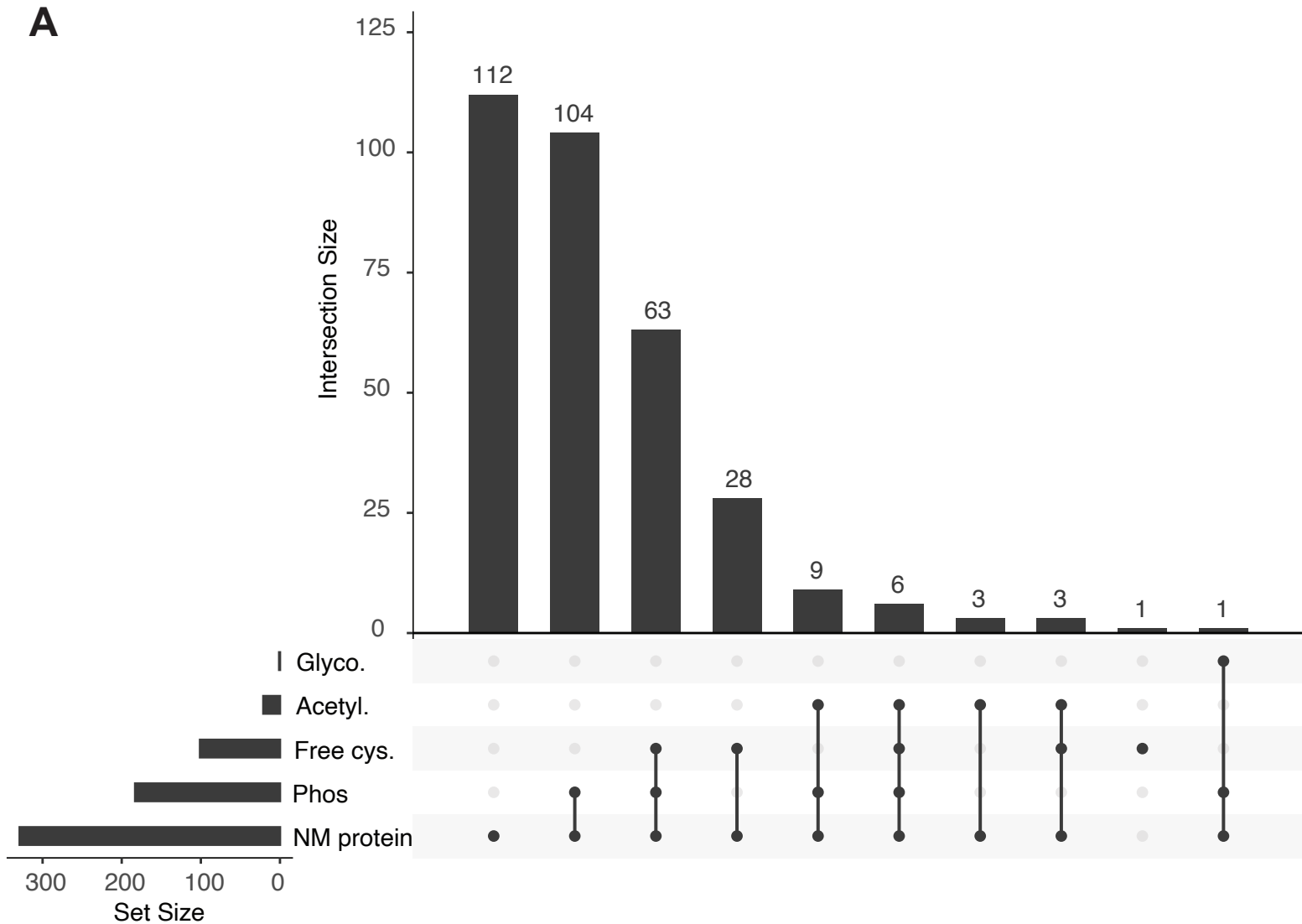**B****Residual distribution by brain region**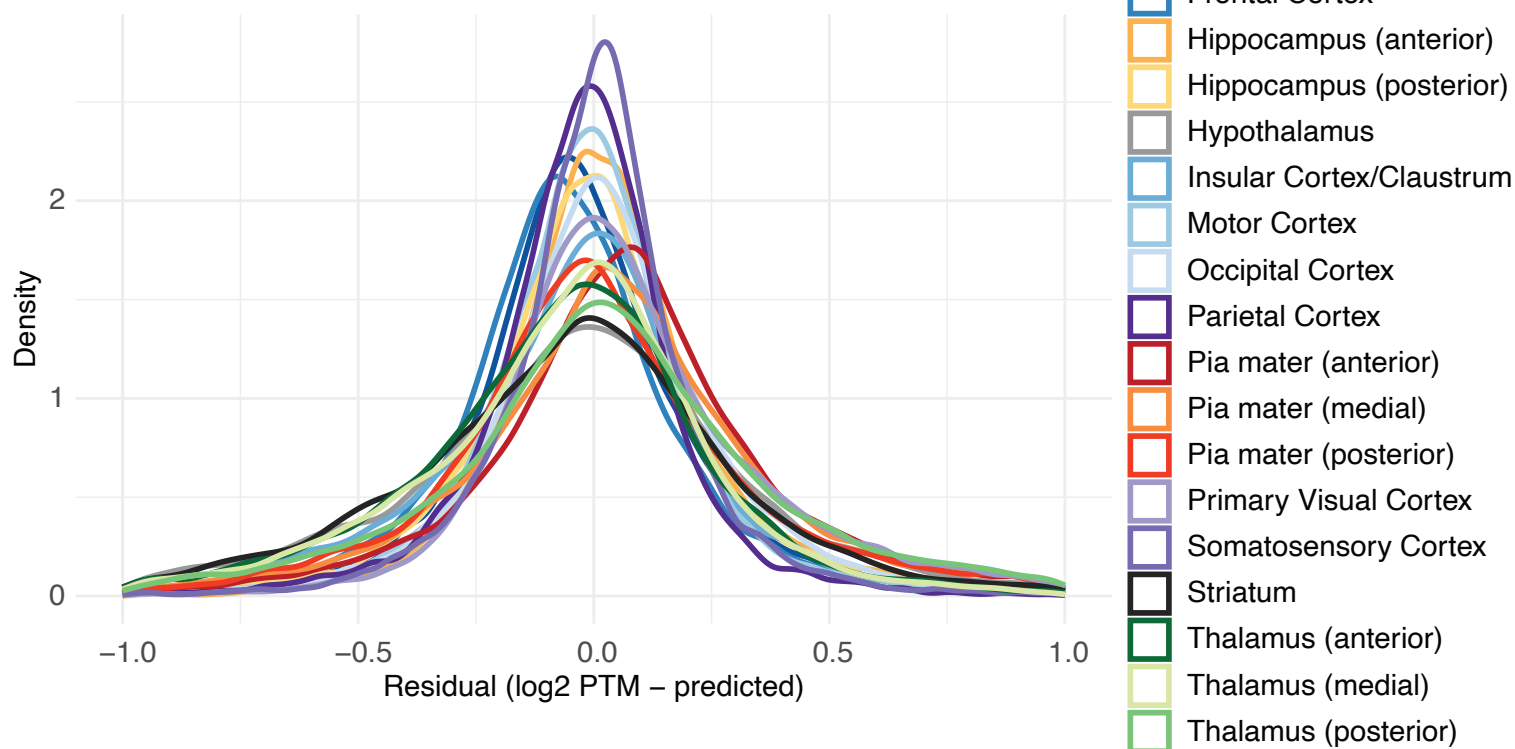

Supplement: Supplementary file 4 — Figure S4. [file JOA-9999-0-s001.pdf]
