# Supplementary material for: A posttranslational proteomic survey of a single anatomically preserved human 20‐week postconception brain
Source: J Anat. 2026 May 3:10.1111/joa.70170. Online ahead of print. doi: 10.1111/joa.70170 (PMC13399142; doi:10.1111/joa.70170)
Supplement: Supplementary file 5 — Figure S5. [file JOA-9999-0-s004.pdf]

Figure S5

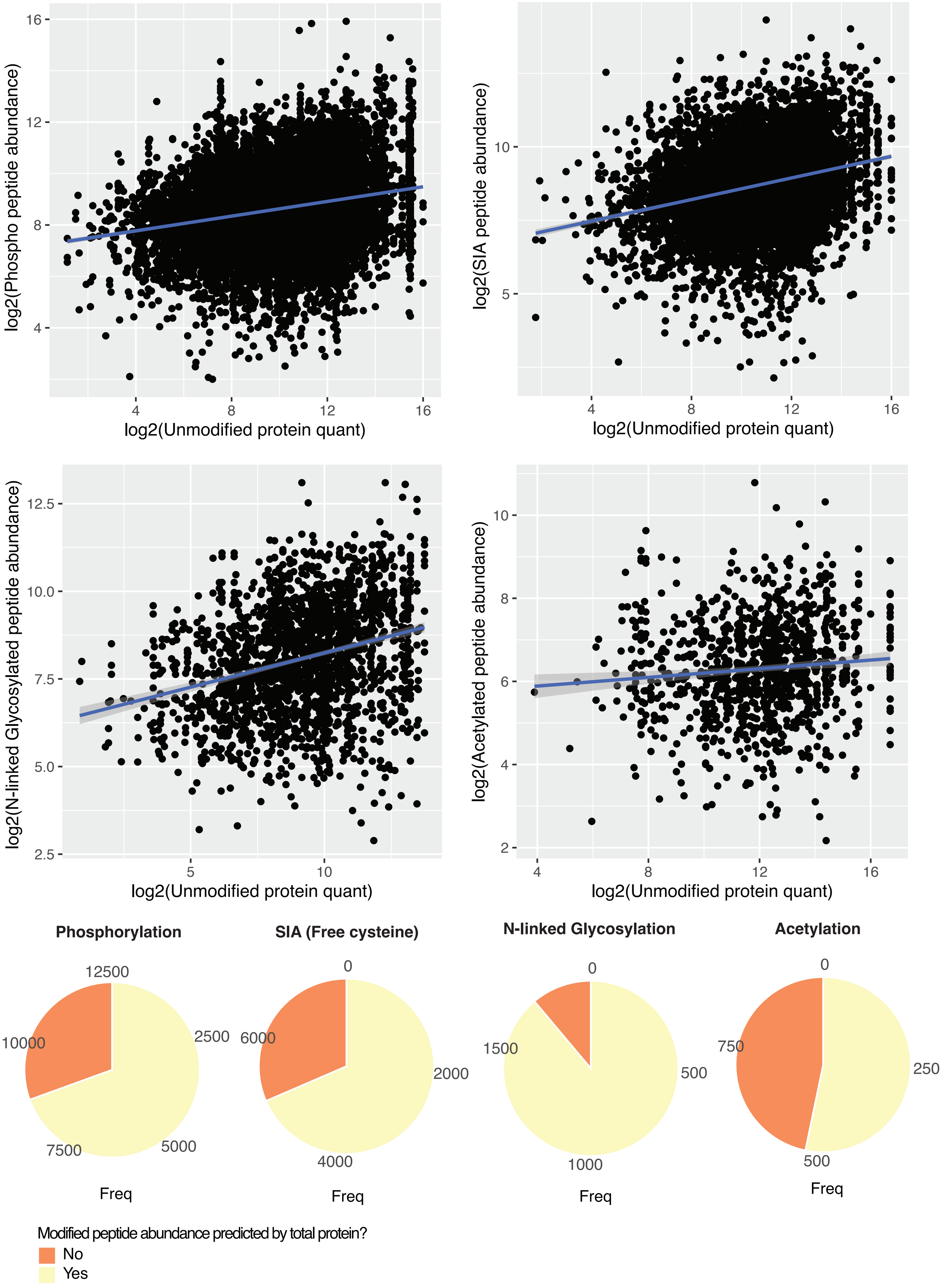
log2(Phospho peptide abundance)
log2(Unmodified protein quant)
log2(SIA peptide abundance)
log2(Unmodified protein quant)
log2(N-linked Glycosylated peptide abundance)
log2(Unmodified protein quant)
log2(Acetylated peptide abundance)
log2(Unmodified protein quant)
Phosphorylation
SIA (Free cysteine)
N-linked Glycosylation
Acetylation
Freq
Freq
Freq
Freq
Modified peptide abundance predicted by total protein?
No
Yes
